# Dynamic colocalization of 2 simultaneously active *VSG* expression sites within a single expression-site body in *Trypanosoma brucei*

**DOI:** 10.1073/pnas.1905552116

**Published:** 2019-07-29

**Authors:** James Budzak, Louise E. Kerry, Aris Aristodemou, Belinda S. Hall, Kathrin Witmer, Manish Kushwaha, Carys Davies, Megan L. Povelones, Jacquelyn R. McDonald, Aakash Sur, Peter J. Myler, Gloria Rudenko

**Affiliations:** ^a^Department of Life Sciences, Imperial College London, SW7 2AZ London, United Kingdom;; ^b^Center for Global Infectious Disease Research, Seattle Children’s Research Institute, Seattle, WA 98109;; ^c^Department of Biomedical Informatics and Medical Education, University of Washington, Seattle, WA 98195;; ^d^Department of Global Health, University of Washington, Seattle, WA 98195

**Keywords:** variant surface glycoprotein, RNA polymerase I, antigenic variation, epigenetics, monoallelic exclusion

## Abstract

Monoallelic exclusion ensures that the African trypanosome *Trypanosoma brucei* exclusively expresses only 1 of thousands of different variant surface glycoprotein (VSG) coat genes. The active *VSG* is transcribed from 1 of 15 polycistronic bloodstream-form VSG expression sites (ESs), which are controlled in a mutually exclusive fashion. Unusually, *T. brucei* uses RNA polymerase I (Pol I) to transcribe the active ES, which is unprecedented among eukaryotes. This active ES is located within a unique extranucleolar Pol I body called the expression-site body (ESB). A stringent restriction mechanism prevents *T. brucei* from expressing multiple ESs at the same time, although how this is mediated is unclear. By using drug-selection pressure, we generated VSG double-expresser *T. brucei* lines, which have disrupted monoallelic exclusion, and simultaneously express 2 ESs in a dynamic fashion. The 2 unstably active ESs appear epigenetically similar to fully active ESs as determined by using chromatin immunoprecipitation for multiple epigenetic marks (histones H3 and H1, TDP1, and DNA base J). We find that the double-expresser cells, similar to wild-type single-expresser cells, predominantly contain 1 subnuclear ESB, as determined using Pol I or the ESB marker VEX1. Strikingly, simultaneous transcription of the 2 dynamically transcribed ESs is normally observed only when the 2 ESs are both located within this single ESB. This colocalization is reversible in the absence of drug selection. This discovery that simultaneously active ESs dynamically share a single ESB demonstrates the importance of this unique subnuclear body in restricting the monoallelic expression of VSG.

## Significance

The African trypanosome *Trypanosoma brucei* expresses a single variant surface glycoprotein (VSG) gene from one of multiple VSG expression sites (ESs) in a stringent monoallelic fashion. The counting mechanism behind this restriction is poorly understood. Unusually for a eukaryote, the active ES is transcribed by RNA polymerase I (Pol I) within a unique Pol I body called the expression-site body (ESB). We have demonstrated the importance of the ESB in restricting the singular expression of VSG. We have generated double-expresser trypanosomes, which simultaneously express 2 ESs at the same time in an unstable dynamic fashion. These cells predominantly contain 1 ESB, and, surprisingly, simultaneous transcription of the 2 ESs is observed only when they are both colocalized within it.

The monoallelic expression of 1 gene out of a large assortment of highly similar variants is a poorly understood phenomenon. The olfactory receptors are the most widely studied example of monoallelic exclusion in mammals, whereby each sensory neuron within the nose expresses 1 odorant receptor gene out of a repertoire of more than 1,400 genes (1, 2). The stochastic but irreversible activation of individual olfactory receptor (OR) genes during development of the olfactory epithelium relies on a 3-node signaling cascade, which locks down the epigenetic state of the chosen OR gene and stabilizes its expression (3, 4). Nuclear architecture plays a key role in the regulation of the OR genes. These form interchromosomal contacts, which result in them clustering in subnuclear OR gene compartments and thereby facilitating monoallelic exclusion (5). Monoallelic exclusion is also vital for parasites controlling expression of multiple similar variant antigen genes. The malaria parasite *Plasmodium falciparum* strictly controls expression of 1 of many *var* genes. Here, nuclear localization also plays a key role, along with other epigenetic mechanisms, including histone modifications and noncoding RNAs (6).

Survival of the African trypanosome *Trypanosoma brucei* relies on monoallelic expression of 1 of thousands of variant surface glycoprotein (VSG) genes. *T. brucei* is a unicellular parasite of the mammalian bloodstream, causing diseases including African sleeping sickness (7). Bloodstream-form *T. brucei* is protected by a dense coat of a single type of VSG (8–10). To ensure that antigenic variation operates properly, it is key that only 1 *VSG* is expressed at a time in each trypanosome. The active *VSG* is located in 1 of ∼15 polycistronic telomeric bloodstream-form *VSG* expression site (BES or ES) transcription units (11). Switching the active *VSG* can involve DNA rearrangements or transcriptional control, as the trypanosome switches between different ESs (12–14). Numerous epigenetic factors including chromatin proteins and remodelers have been identified, which ensure that a particular ES activation state is maintained (14–18). However, very little is known about how ESs are rigorously counted in such a stringent fashion.

Unusually, the protein-coding genes within the *T. brucei* ESs are transcribed by RNA polymerase I (Pol I), which is unprecedented among eukaryotes (19, 20). Typically, Pol I exclusively transcribes ribosomal DNA (rDNA) in a subnuclear Pol I body known as the nucleolus (21). However, in addition to the nucleolus, there is a unique nonnucleolar Pol I body in *T. brucei* known as the expression-site body (ESB), which contains the active ES (22, 23). Very little is known about the architecture of the ESB. The VEX1 protein has been identified as uniquely locating to the ESB, but its exact function is still unclear (24). In other organisms that use monoallelic exclusion, nuclear architecture plays a role in controlling the activation and silencing of allelic loci (5, 25). However, in *T. brucei*, the influence of nuclear positioning on the monoallelic exclusion of the ESs is still poorly understood (17).

To better understand the restrictions operating on monoallelic exclusion, we generated trypanosome cell lines in which this was disrupted. Although bloodstream-form *T. brucei* expresses only a single ES at a time, perturbation of ES monoallelic exclusion can be achieved by using drug-selection pressure (26, 27). We used this approach to generate independently derived *T. brucei* double-expresser strains that unstably and transiently express 2 ESs at the same time. These unstably transcriptionally active ESs appear to have an epigenetic state similar to that of fully active ESs. We find that the 2 ESs are in closer proximity to each other when they are dynamically transcribed in double-expresser *T. brucei* compared with when only 1 ES is active in single-expressers. The double-expresser *T. brucei* typically have a single ESB, similar to single-expressers. Strikingly, we see simultaneous transcription of 2 ESs only when these are colocalized within a single ESB. These results showing that the 2 dynamically transcribed ESs appear to continuously transition between a single shared ESB demonstrate the importance of this unique subnuclear body in restricting the monoallelic expression of VSG.

## Results

**Fig. 1.**
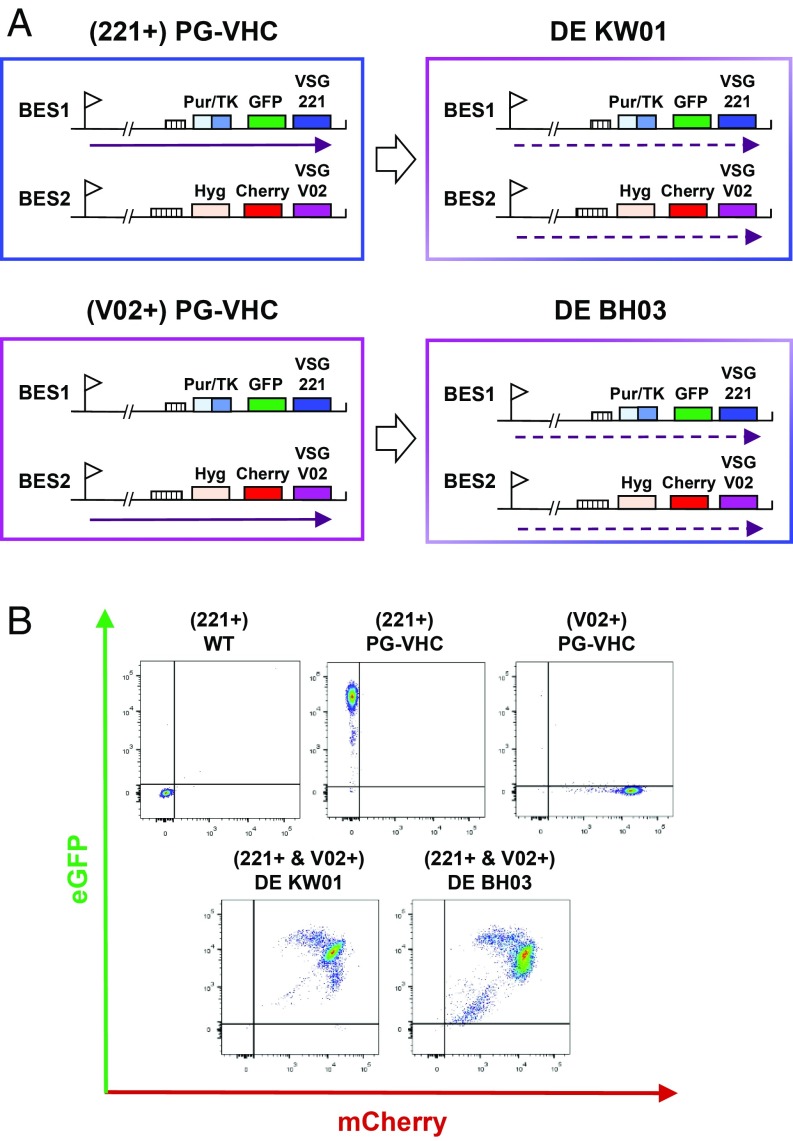
Selection for simultaneous activation of 2 *VSG* expression sites results in the generation of *T. brucei* double-expresser (DE) cell lines. (*A*) The schematics indicate the parental cell line (221+)PG-VHC *VSG221* with an active *VSG221* expression site (BES1) and the (V02+)PG-VHC line with an active *VSGV02* expression site (BES2). In both cell lines, the puromycin/thymidine kinase fusion (Pur/TK) and *GFP* genes are inserted upstream of *VSG221* in BES1, and the hygromycin resistance (Hyg) and mCherry genes are inserted upstream of *VSGV02* in BES2. The double-expresser (DE) DE KW01 cell line was generated by using drug selection for activation of both ESs using the (221+)PG-VHC strain, and the DE BH03 line was generated by using the same approach with the parental (V02+)PG-VHC line (large open arrows). The ES promoters are indicated with flags, transcription with arrows, characteristic 70-bp repeats with hatched boxes, and the relevant genes with colored boxes. Dynamic ES transcription is indicated with arrows with dashed lines. (*B*) Double-expression of VSG expression sites monitored by flow cytometry. Traces show quantitation of GFP expressed from BES1 on the *y* axis and mCherry expressed from BES2 on the *x*-axis. (*Top*) Nonfluorescent wild type (WT) VSG221 (221+)-expressing strain compared with the VSG221 and VSGV02 expressing parental lines (221+)PG-VHC or (V02+)PG-VHC. (*Bottom*) Traces from the 2 double-expresser lines DE KW01 and BH03, which express both BES1 and BES2 at the same time (221+ & VO2+). Gates are shown as 98th percentile relative to the control.

To select for simultaneous activation of 2 ESs, we generated *T. brucei* strains with drug resistance genes inserted into the telomeric regions of BES1 and BES2 (11). We first inserted the eGFP and puromycin/thymidine kinase (TK) genes immediately upstream of *VSG221* in BES1 (Fig. 1*A*) (11). Using TK as a negative selectable marker, we subsequently generated *VSG* switch variants, of which 1 had activated BES2 (11). We next inserted the hygromycin resistance and mCherry genes upstream of *VSGV02* in BES2 to generate the VSGV02-expressing (V02+)PG-VHC line (Fig. 1*A*). Selection for reactivation of BES1 resulted in generation of the VSG221-expressing (221+)PG-VHC line.

Both parental lines were subjected to drug-selection pressure to select for simultaneous activation of both BES1 and BES2. We used concentrations of selection drugs that are higher than standard concentrations to increase the stringency of selection, as this did not have a deleterious effect on growth (*SI Appendix*, Fig. S1 *A* and *B*). We generated the double-expresser DE KW01 line from the BES1-expressing (221+)PG-VHC parental line, and the DE BH03 line from the BES2-expressing (V02+)PG-VHC line (Fig. 1*A*). Frequency of generation of double-expresser (DE) lines was very low (<10^−8^). The growth rate of the DE KW01 line was equivalent to the parental line, whereas the DE BH03 line appeared to show a minor reduction in growth rate (*SI Appendix*, Fig. S1*C*).

To confirm that both marked ESs in the double-expresser cells were active, we used flow cytometry to monitor ES activation state. As expected, the parental (221+)PG-VHC line expressed GFP, and the (V02+)PG-VHC line expressed mCherry (Fig. 1*B*). In contrast, the double-expresser DE KW01 and DE BH03 cell lines expressed both fluorescent proteins at the same time (Fig. 1*B*), and expression of a fluorescent protein gene within a given ES corresponded with expression of the respective VSG (*SI Appendix*, Figs. S2–S4). Both double-expresser lines expressed approximately equivalent levels of VSG221 and VSGV02, each at approximately half of normal VSG levels, as monitored by Western blot analysis (*SI Appendix*, Fig. S2). As expected, both VSG221 and VSGV02 appeared to show normal cellular localization (*SI Appendix*, Fig. S3).

**Fig. 2.**
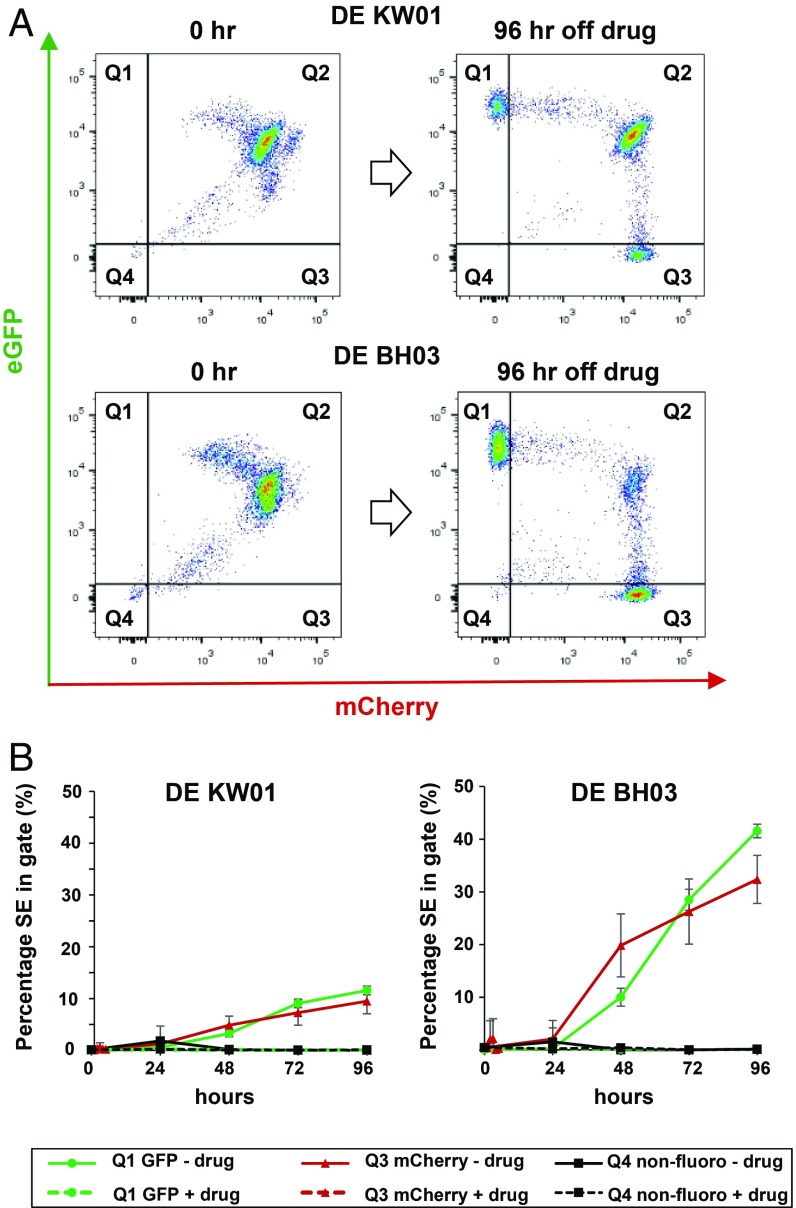
Removal of double-expresser strains from double drug-selection pressure results in loss of the double-expresser (DE) phenotype with strain-dependent kinetics. (*A*) Double-expresser lines DE KW01 and DE BH03 were removed from drug selection for the time indicated in hours (h). Representative flow cytometry traces are shown with quadrants containing populations positive for eGFP (Q1), mCherry (Q3), both eGFP and mCherry (Q2), or nonfluorescent (Q4). Quadrants are based on 98th percentile gating. (*B*) Quantitation of the percentage of single-expressers (cells in quadrants Q1 or Q3) present in populations of DE KW01 or DE BH03 after removal of drug selection. Values shown are the average ± standard deviation (SD) of 3 biological replicates.

The 2 parental (221+)PG-VHC and (V02+)PG-VHC lines expressed the relevant active ES even in the absence of drug-selection pressure for 96 h (*SI Appendix*, Fig. S5). Only a minor fraction of trypanosomes were nonfluorescent, possibly as a consequence of a switch to another ES not containing a fluorescent protein gene. However, removal of the double-expresser DE KW01 and DE BH03 strains from drug-selection pressure resulted in a process of relapse back to single expression of either BES1 or BES2 with strain-dependent kinetics (Fig. 2). The DE BH03 strain reverted back to single-expression relatively quickly compared with the DE KW01 strain, which was more stable. Possibly, these strains have acquired different genetic mutations, which impact their ability to establish a double-expresser state. Both double-expresser lines started to revert back to either BES1 or BES2 in a relatively unbiased manner, irrespective of the ES expressed by the original parental line from which the double-expresser was derived.

We did not see significant numbers of nonfluorescent trypanosomes even after 96 h off drug selection (gate Q4; Fig. 2). This indicates that the trypanosomes switched back to either BES1 or BES2, rather than activating 1 of the 13 silent ESs. This implies that the epigenetic state of the unstably transcriptionally active ESs in double-expresser cells can be heritably transmitted over many days rather than being rapidly erased. The extent of these putative epigenetic modifications appeared to be sufficient to enable the rapid and full reactivation of the 2 unstably active ESs.

**Fig. 3.**
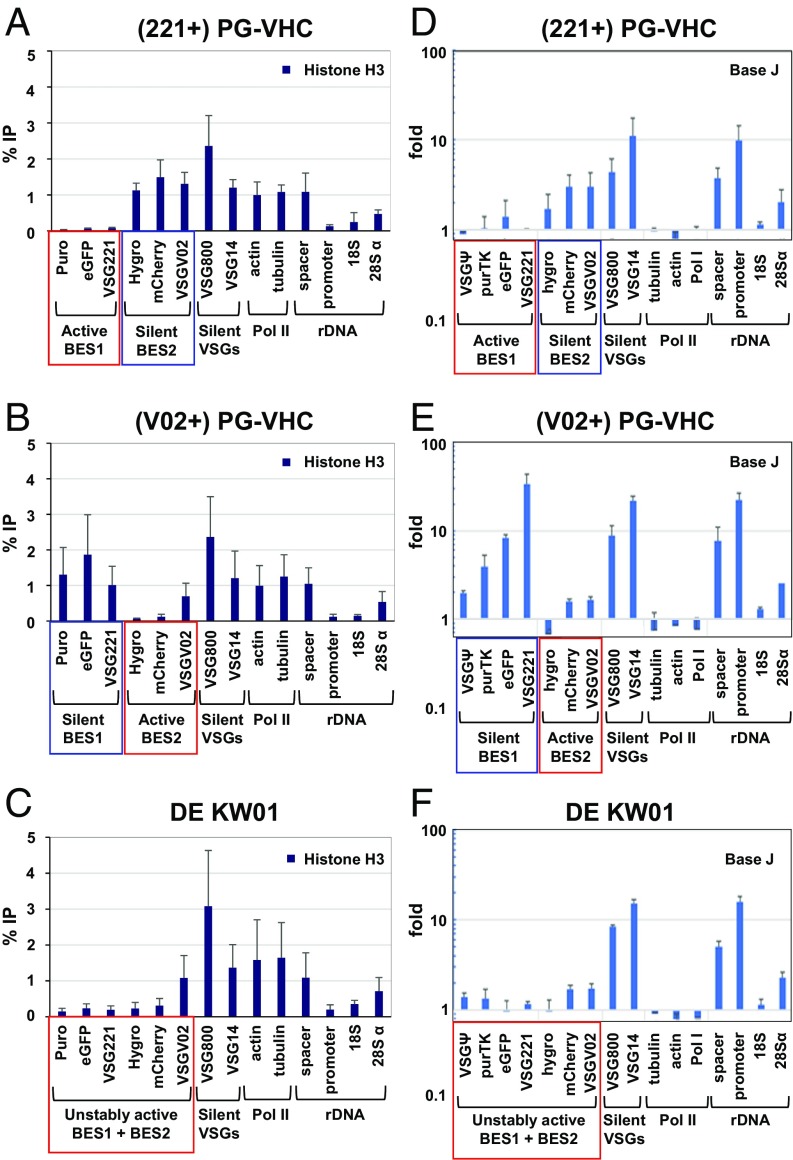
The unstably active ESs in the double-expresser DE KW01 strain have similar epigenetic features to the active ES in the parental single-expresser lines. The genes analyzed are indicated below, and include loci in the Pol I transcribed ESs and rDNA compared with Pol II transcription units. Data from the active ESs are indicated with red boxes and silent BESs with blue boxes. (*A*–*C*) The distribution of histone H3 was determined by using ChIP-qPCR in the single-expresser (221+)PG-VHC (*A*) or (V02+)PG-VHC (*B*) lines compared with the double-expresser DE KW01 line (*C*). Data are shown as the percentage of input immunoprecipitated (% IP) after subtraction of the no-antibody control. The mean of 3 independent ChIP-qPCR experiments is shown, with SD indicated with error bars. (*D*–*F*) The distribution of base J was determined by using J-IP-seq in the parental (221+)PG-VHC (*D*) or (V02+)PG-VHC (*E*) lines compared with DE KW01 (*F*). Fold enrichment was calculated for each gene as the ratio of median-normalized count in the J-IP library to the corresponding pre-IP library.

To investigate this further, we determined the distribution of various epigenetic markers at different activation states of BES1 and BES2. The inserted fluorescence protein and drug resistance genes served as single copy sequences in addition to the VSG genes, allowing the unambiguous differentiation of BES1 and BES2 from the 13 other highly similar ESs in *T. brucei* 427 (11). When transcriptionally active, the Pol I transcribed ESs are unusually depleted of histones (28–30), which is characteristic of actively transcribed Pol I transcription units in general (31, 32). By using chromatin immunoprecipitation (ChIP) of the core histone H3, we established that, as expected, the parental (221+)PG-VHC and (V02+)PG-VHC cell lines showed greatly reduced levels of histone H3 on the 1 fully active ES compared with a silent ES (Fig. 3 *A* and *B*). In contrast, the double-expresser DE KW01 line showed depleted levels of histone H3 on both the unstably active BES1 and BES2. These levels were comparable with those on fully active ESs (Fig. 3*C*). These ChIP results were particularly clear when single copy genes were analyzed (eGFP *P* < 0.01, mCherry *P* < 0.05). However, in the case of VSGV02, a second silent copy of the VSGV02 gene not located within BES2 could also have given rise to the ChIP signal. The same relative histone depletion on active ESs was observed in ChIP experiments detecting the linker histone H1 (*SI Appendix*, Fig. S6) (30).

An additional epigenetic mark distinguishing ESs according to activation state is the glucosylated DNA nucleotide base J (33). Base J is enriched at the telomeres of the silent ESs, with the concentration of base J increasing in a gradient extending toward the telomere end (34). By using an antibody against base J (34) in ChIP-seq experiments (35), we found that levels of base J-containing DNA indeed increased in a gradient extending toward the telomere of a silent ES in the single-expresser lines (Fig. 3 *D* and *E*). In contrast, base J levels were very low in the active ES (Fig. 3 *D* and *E*). In the double-expresser DE KW01 line, base J levels were highly reduced at the unstably active BES1 and BES2, comparable to an active ES (eGFP *P* < 0.01, VSG221 *P* < 0.0001; Fig. 3*F*). The architectural chromatin protein TDP1, in contrast to histones and base J, is enriched on active ESs and depleted on silent ESs (36, 37). Here too, TDP1 distribution over the unstably active BES1 and BES2 appeared to be comparable to that observed on fully active ESs (*SI Appendix*, Fig. S7). Although the results of the TDP ChIP were not statistically significant, they indicate a trend. All of these data are compatible with the unstably active BES1 and BES2 having the epigenetic marks and chromatin state of fully active ESs. This active chromatin state possibly facilitates the rapid switching between the 2 selected ESs, thereby enabling the double-expresser phenotype.

**Fig. 4.**
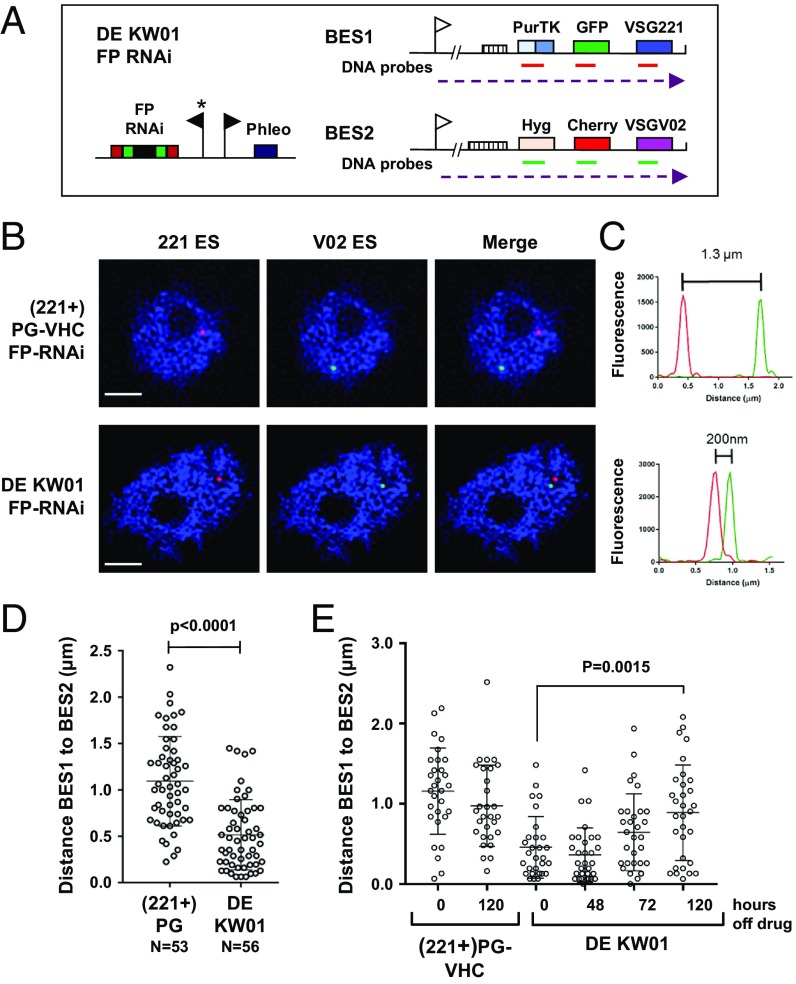
ESs are located in closer proximity to each when there is selection for simultaneous activation in a double-expresser strain compared with the single-expresser parental strain. (*A*) The schematic shows the DE KW01 FP-RNAi cell line, which contains the FP-RNAi construct, which allows transient knock-down of the fluorescent proteins eGFP and mCherry. Promoters are indicated with flags, and the tetracycline-inducible promoter indicated with an asterisk. Relevant genes are indicated with colored boxes and unstable transcription of BES1 and BES2 with dashed arrows. The location of the DNA-FISH probes for BES1 are indicated with red lines and those for BES2 with green lines. (*B*) Representative superresolution microscopy images of DNA FISH performed in the single-expresser (221+)PG-VHC FP-RNAi and double-expresser DE KW01 FP-RNAi lines after probing for BES1 (red) and BES2 (green). DNA is stained with DAPI (blue). (Scale bars, 1 µm.) (*C*) Quantitation of the distance between BES1 and BES2 in the images shown in *B*. Fluorescence intensity is shown in arbitrary units. (*D*) Distance between BES1 and BES2 in the single-expresser (221+)PG-VHC cell line compared with the double-expresser DE KW01 line as determined by using DNA FISH and superresolution microscopy. Data were collected from at least 3 biological replicates, with the average value shown with a bar ± SD and the significance of colocalization indicated with the *P* value. Total number (N) of cells analyzed is indicated below. (*E*) Reversibility of BES colocalization shown through quantitation of the distance between BES1 and BES2. DNA FISH was performed in the (221+)PG-VHC and DE KW01 cell lines in the presence or after the removal of drug selection for the time indicated in hours. Data were collected at 0 h, 24 h, 48 h, 72 h, 96 h, and 120 h for both the (221+)PG-VHC and DE KW01 cell lines; however, only some relevant time points are shown. Data shown were obtained from 3 biological replicates by using immunofluorescence microscopy, with at least 29 cells quantitated per time point. The average value is indicated ± SD with the significance of reversibility of colocalization indicated with a *P* value.

We next investigated the subnuclear location of BES1 and BES2 in the double-expresser DE KW01 strain compared with its single-expresser parent (221+)PG-VHC using structured illumination superresolution microscopy (SIM-SR). As these cell lines fluoresce red and green, we first inserted an inducible RNAi construct targeting both eGFP and mCherry into these lines (Fig. 4*A*). After inducing fluorescent protein (FP) RNAi for 72 h, the resulting cell lines were not fluorescent. This allowed us to perform DNA fluorescence in situ hybridization (FISH) experiments using probes specific for either BES1 or BES2.

In the parental (221+)PG-VHC FP-RNAi cells, only BES1 is transcriptionally active. DNA probes specific for either BES1 or BES2 allowed identification of these ESs by FISH (Fig. 4*B*). BES1 and BES2 were normally not in close proximity to each other (Fig. 4*C*), and were relatively randomly distributed within the nucleus, with an average distance of 1.1 ± 0.49 µm (Fig. 4*D*). In contrast, in the double-expresser DE KW01 FP-RNAi cells in which both BES1 and BES2 are unstably transcriptionally active, BES1 and BES2 were in significantly closer proximity to each other. They were separated with an average distance of 0.5 ± 0.38 µm (*P* < 0.0001), with 25% located within 200 nm of each other (Fig. 4*D*).

This preferential colocalization of BES1 and BES2 in the double-expresser DE KW01 strain was reversible in the absence of double drug selection. If double-expresser DE KW01 cells were removed from drug selection for 120 h, the average distance between BES1 and BES2 increased significantly from 0.45 ± 0.38 µm to 0.89 ± 0.6 µm (*P* < 0.0015; Fig. 4*E*). This average distance between BES1 and BES2 is comparable to that observed in the single-expresser (221+)PG-VHC line. This observation that BES1 and BES2 moved apart from each other in the absence of drug selection was coincident with reversion of the cells back to a single-expresser state, as was shown earlier by using flow cytometry (Fig. 2*A*).

**Fig. 5.**
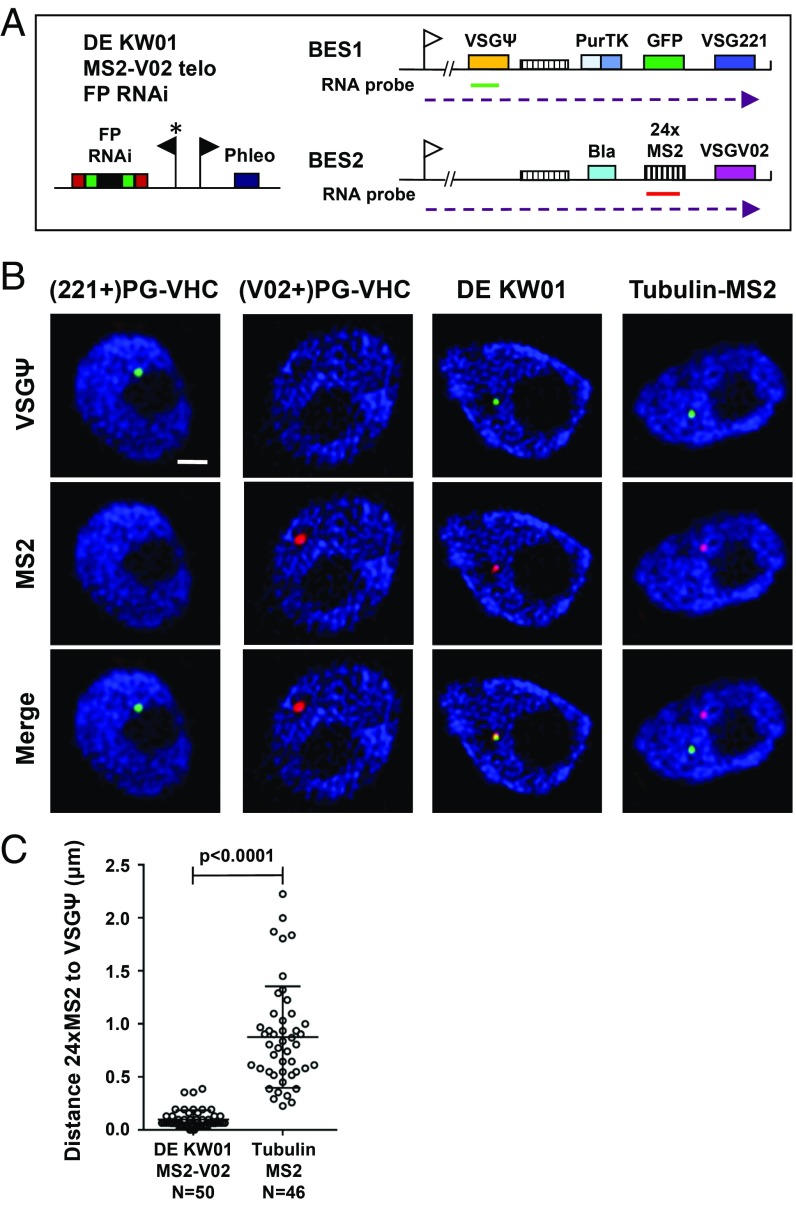
Simultaneous transcription of 2 unstably active ESs in the same subnuclear location in double-expresser DE KW01 cells. (*A*) Schematic of the DE KW01 MS2-V02 telo FP-RNAi cell line used for the RNA FISH experiments. This line contains a construct with a 24× MS2 repeat cassette linked to a blasticidin resistance gene (bla) integrated immediately upstream of the *VSGV02* gene. Stellaris RNA FISH probes are indicated, with 1 set hybridizing with the VSGΨ in BES1 (green line) and the other set hybridizing with the MS2 repeat sequence in BES2 (red line). Further, the schematic is as described in Fig. 4*A*. (*B*) Representative superresolution microscopy images of RNA FISH performed on the single-expresser (221+)PG-VHC or (V02+)PG-VHC cell lines compared with the double-expresser DE KW01. A cell line containing MS2 repeats in the tubulin locus (Tubulin-MS2) is included as a control. Experiments using probes against the VSGΨ (green) or the 24× MS2 repeats (red) are shown, with DNA stained with DAPI (blue). (Scale bar, 500 nm.) (*C*) Quantitation of the distance between nascent VSGΨ and MS2 repeat containing transcripts using superresolution microscopy. The MS2 repeats are located in BES2 in DE KW01 MS2-V02 or in the tubulin locus (Tubulin-MS2). The average distance is shown ± SD with the significance of colocalization indicated with a *P* value.

We next performed Stellaris RNA FISH to determine where transcription of the 2 unstably active ESs was occurring. Induction of RNAi against the fluorescent proteins resulted in the cell lines becoming nonfluorescent so that RNA FISH could be performed. BES1 transcription was then detected with a probe for a single copy VSG pseudogene (VSGΨ), which is transcribed into an unstable nuclear RNA transcript. To detect BES2 transcription, a construct containing 24 MS2 repeats was inserted into the BES2 telomere in the single-expresser (V02+)PG-VHC and double-expresser DE KW01 FP-RNAi strains (Fig. 5*A*). The MS2-containing sequences are also transcribed into an unstable nuclear RNA that can be detected by RNA FISH.

As expected for probes detecting unstable ES RNA transcripts, RNA signal was detected exclusively in the nucleus by using superresolution microscopy. Transcription of BES1 was detected in the (221+)PG-VHC strain using the VSGΨ probe, or from BES2 in the (V02+)PG-VHC strain using the MS2 repeat probe (Fig. 5*B*). However, strikingly, in cells in which simultaneous transcription from BES1 and BES2 was observed by using RNA-FISH, transcripts from both BES1 and BES2 (detected using VSGΨ or MS2 probes) colocalized within 200 nm of each other (0.098 ± 0.085 µm) in 94% of the cells (Fig. 5*C*). In fact, in the majority (72%) of DE KW01 cells with simultaneous BES1 and BES2 transcription, these transcripts were located within 100 nm of each other (Fig. 5*C*). In contrast, transcripts from MS2 repeats that had been inserted into the tubulin arrays in the Tubulin-MS2 cell line were not in close proximity to transcripts from BES1 (0.88 ± 0.48 µm; *P* < 0.0001; Fig. 5*C*).

**Fig. 6.**
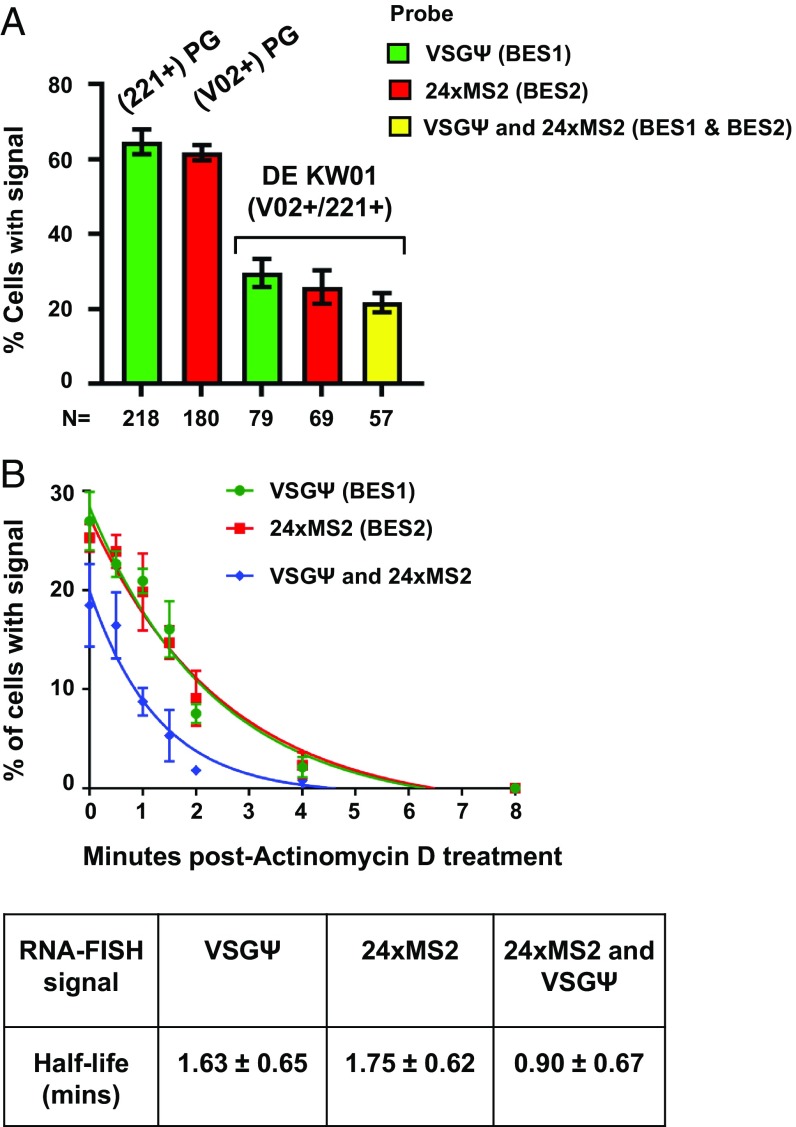
Transcription of unstably active ESs in double-expresser DE KW01 cells. (*A*) Quantitation of the percentage of cells with transcripts detectable from BES1 (VSGΨ), BES2 (24× MS2 repeats), or both BES1 and BES2 in the single-expresser (221+)PG-VHC and (V02+)PG-VHC strains or the double-expresser DE KW01 line. Data were obtained from RNA FISH experiments performed on G1 cells analyzed using superresolution microscopy. Values shown are the averages ± SD from 3 biological replicates with total number of cells (N) indicated below. (*B*) Half-life of the RNA FISH signal from BES1 (VSGΨ) or BES2 (24× MS2 repeats) transcription foci in the DE KW01-MS2-V02 cell line following treatment with sinefungin and actinomycin D. Half-lives of each RNA FISH signal in minutes (min) are shown in the table. The values shown are the averages ± SD of 3 biological replicates, with at least 300 cells counted per time point.

In agreement with the highly dynamic nature of ES transcription in the double-expresser DE KW01 line, RNA FISH experiments showed that BES1 and BES2 were not simultaneously transcribed in all cells (Fig. 6*A*). In the single-expresser (V02+)PG or (221+)PG lines, transcript was detected for either BES1 or BES2 in ∼62 to 65% of the cells (Fig. 6*A*). However, in the double-expresser KW01 line, 30% of the cells exclusively contained BES1 transcript (VSGΨ), 26% of the cells contained only BES2 transcript (24× MS2), and 22% of the cells simultaneously contained transcript specific for both BES1 and BES2. Transcripts were not detectable for either ES in 22% of the cells.

To obtain better insight into this dynamic ES transcription, we determined the half-lives of both VSGΨ (BES1) and 24× MS2 repeat-containing transcripts (BES2) in DE KW01. We blocked transsplicing by using sinefungin treatment, followed by blocking transcription with actinomycin D (38). Visualization of both ES-specific transcripts by microscopy showed that they had very short half-lives of 1.63 ± 0.65 min (VSGΨ) or 1.75 ± 0.62 min (24× MS2 repeat). Simultaneous detection of both transcripts disappeared with a half-life of 0.90 ± 0.67 min (Fig. 6*B*). These results all support a model in which there is very rapid and dynamic switching between transcription of BES1 and BES2 in double-expresser DE KW01 cells. Simultaneous presence of both BES1 and BES2 nascent transcripts within the same cell normally occurs only if both ESs are juxtaposed within 200 nm of each other in the same subnuclear location.

**Fig. 7.**
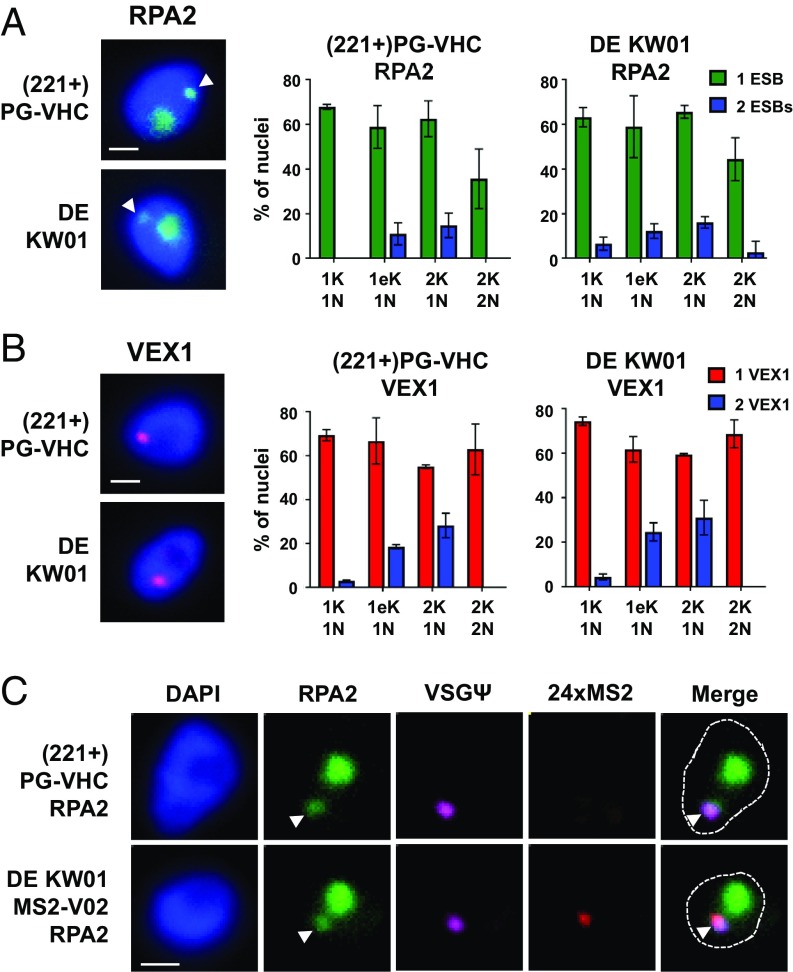
Double-expresser DE KW01 cells predominantly contain a single Pol I and VEX1 focus, which is the location of transcription of the simultaneously active ESs. (*A*) Immunofluorescence microscopy analysis of single-expresser (221+)PG-VHC or double-expresser KW01 cells expressing the Pol I subunit RPA2 tagged with mNeonGreen (mNG::RPA2). The extranucleolar ESB is indicated with an arrowhead. The percentage (%) of nuclei containing 1 or 2 ESB foci is indicated for cells at different stages of the *T. brucei* cell cycle, with the number of kinetoplasts (K), elongated kinetoplasts (eK), or nuclei (N) indicated below. Values shown are the average ± SD of 3 biological replicates. The number of cells counted is greater than or equal to 300, 70, 50, and 30 nuclei for the 1K1N, 1eK1N, 2K1N, or 2K2N stages, respectively. (Scale bars: 1 µm.) (*B*) As in *A*, only the single-expresser (221+)PG-VHC or double-expresser KW01 cells have VEX1 tagged with the 12× myc epitope. (*C*) Fluorescence microscopy analysis of epitope-tagged Pol I subunit RPA2 mNG::RPA2 (green) together with RNA FISH probing for VSGΨ (magenta) or 24× MS2 repeat (red) nascent transcripts in the single-expresser (221+)PG-VHC or double-expresser DE KW01 lines containing mNG::RPA2. DNA is stained with DAPI, with the nucleus indicated with a dotted line and the ESB with a white arrowhead. (Scale bars, 1 µm.)

Based on these FISH data, we hypothesized that the 2 unstably active ESs were sharing a single ESB. We therefore determined the number of ESBs in the double-expresser DE KW01 compared with the single-expresser (221+)PG-VHC strain. The ESB can be identified by using Pol I and VEX1 as markers (22, 24). We tagged the RPA2 subunit of Pol I with mNeonGreen (mNG) and the VEX1 ESB marker with a 12×myc epitope in both the single-expresser and double-expresser strains (24). In strains that contain epitope-tagged Pol I, a small extranucleolar focus can be seen that corresponds to the ESB (Fig. 7*A*, white arrowhead), which is in addition to the nucleolus (Fig. 7*A*). Quantitation of Pol I foci throughout the cell cycle showed that a similar distribution is observed in both the single-expresser (221+)PG-VHC and double-expresser DE KW01 strains, with cells predominantly containing a single ESB. Similar results were obtained if the ESB marker VEX1 was monitored. Here also, a single VEX1-containing ESB focus was typically seen in the cell, with a similar distribution of VEX1 foci observed in both single-expresser and double-expresser cells (Fig. 7*B*).

We next determined where simultaneous transcription of BES1 and BES2 was occurring with relation to the ESB in the double-expresser cells. Following RNAi-mediated knockdown of GFP and mCherry, we performed RNA FISH visualizing BES1 transcription using a VSGΨ probe and BES2 transcription using a probe for the 24× MS2 repeats in cells in which the RPA2 Pol I subunit had been epitope-tagged with mNeonGreen (Fig. 7*C*). When both BES1 and BES2 were simultaneously transcribed in the double-expresser DE KW01 cells in the G1 stage of the cell cycle, the BES1-specific transcript (VSGΨ) and the BES2-specific transcript (24× MS2) transcript normally both colocalized within 1 extranucleolar Pol I focus (83% of cells; Fig. 7*C* and *SI Appendix*, Fig. S8*A*). This argues that the presence of a single Pol I-containing ESB restricts the monoallelic expression of VSG. In the small number of cells that had 2 Pol I foci (6.5%), most had BES1 transcript (VSGΨ) in 1 focus and BES2 transcript (MS2) in the other (*SI Appendix*, Fig. S8*B*). We did not observe cells with both BES transcripts in a single Pol I focus when 2 ESBs were present. In cells in which transcriptional signals were simultaneously detected from both BES1 and BES2, the individual BES signals were reduced compared with signals from cells in which either BES1 or BES2 were transcribed individually (*SI Appendix*, Fig. S8*B*). This argues that the ESB contains limiting factors for ES transcription, and the restriction of ES transcriptional components to a single ESB facilitates monoallelic exclusion. Our results highlight the importance of a single ESB structure in restricting the monoallelic expression of VSG.

## Discussion

We have selected for simultaneous activation of 2 ESs in double-expresser *T. brucei* strains, and show that these 2 ESs are transcribed in a highly dynamic and unstable fashion (26). These transiently transcriptionally active ESs appear to have the epigenetic state of fully active ESs, as monitored using epigenetic markers including histones H3 and H1, the DNA modification base J, and the architectural chromatin protein TDP1. Under selection, the 2 ESs migrated within closer proximity of each other within the nucleus. However, strikingly, simultaneous ES transcription was normally observed only when the 2 ESs were within 200 nm of each other. This privileged location corresponds to the ESB subnuclear compartment, which is characterized by the presence of Pol I and VEX1. The 2 selected ESs do not appear able to simultaneously occupy a single ESB in a stable fashion. As a consequence, a highly dynamic transcriptional state is generated, as both ESs alternate back and forth between a single shared ESB and only temporarily occupy the same ESB. These results argue that the ESB is the key component that restricts monoallelic exclusion within African trypanosomes.

The ESB is a non–membrane-bound nuclear body similar to nucleoli and Cajal bodies (17, 39–41). Formation of both of these cellular bodies requires RNA transcription, in a process of phase transition-driven nuclear body assembly (22, 39–45). The formation of the ESB also appears to be transcription-nucleated, as blocking Pol I transcription by using Pol I transcriptional inhibitors results in rapid ESB disassembly, as monitored by using Pol I and the ESB marker VEX1 (46).

If the ESB (similar to the nucleoli) is seeded by RNA, why do 2 ESBs not normally form when selection for 2 active ESs is applied? ES transcription initiation on its own does not appear to be sufficient for nucleation of a functional ESB. It has been shown that, despite high levels of transcription from the active ES, promoter-proximal ES transcripts are also generated from multiple additional ES promoters (47, 48). However, these ES transcripts are not polyadenylated, arguing that these partially active ESs do not have access to efficient RNA processing (48). The ESB structure must therefore contain both transcription and RNA-processing machinery. If these ESB components are limiting within the nucleus and naturally coalesce with each other to self-organize into a single ESB, in a “winner-takes-all” model, the equilibrium would favor 1 functional ESB (24). This restriction would therefore ensure that the 2 active ESs in our double-expresser cells would predominantly share a single ESB. This model requires self-assembly of the ESB in a dynamic fashion.

Although most double-expresser cells contain 1 ESB, this is not always the case. In a small subset of double-expresser cells in G1 which simultaneously contain BES1 and BES2 transcripts, 2 ESBs (as characterized by Pol I) were present rather than 1. However, the relative rarity of this event argues that this configuration is not stable. Possibly, the trypanosome is capable of forming 2 functional ESBs within the same cell, with each ESB occupied by a different ES. However, the nascent transcripts from these 2 different ESs (although not colocalizing) were in relative proximity to each other. This argues that the second ESB might have split off from the first rather than being independently generated. The fact that this configuration of 2 ESBs within 1 cell was found so infrequently argues that this state may be energetically unfavorable. It has been proposed that non–membrane-enclosed nuclear bodies naturally coalesce and self-assemble on RNA within the nucleus in a manner similar to liquid droplets, as this is energetically favorable (49, 50). This model would explain why multiple ESBs would eventually fuse into 1.

If 2 ESBs cannot be stably formed, why do the 2 selected ESs in double-expressers not stably occupy a single ESB? It is likely that the ESB has a defined architecture that is optimized for high levels of transcription and processing of transcripts from a single ES. When 2 ESs are selected for, as both ESs appear to have the epigenetic marks of fully active ESs, the cell cannot discriminate which one should occupy the ESB. The 2 ESs could therefore be in competition with each other for components of the ESB. That competition results in a dominant ES, which temporarily hijacks the entire structure. This process is possibly what occurs during an in situ switch event when an invading ES is activated by taking over the ESB.

If the cell normally only self-assembles a single stable ESB, this makes it unclear if the presence of both BES1 and BES2 transcripts in the same ESB indeed documents simultaneous transcription of both ESs. Instead, this could be the result of rapid alternating transcriptional activity of the 2 ESs. The relatively short half-lives of the nascent ES transcripts (<1 min for both BES1 and BES2 transcripts combined) argues that the 2 ESs might be very rapidly switching back and forth between a single ESB compartment.

The highly dynamic nature of these ES interactions is indicated by the results of the DNA FISH experiments. Although the 2 ESs investigated are located relatively randomly (within 1.1 ± 0.49 µm of each other) in wild-type cells, this proximity is reduced to 0.5 ± 0.38 µm in the presence of simultaneous selection. However, it is only when the 2 ESs are located within 200 nm of each other within a single ESB that transcripts from both ESs are observed in 94% of the cells. This argues that the 2 selected ESs need to be in close enough proximity to each other to allow the invading ES to hijack ESB components from the first ES. This appears to occur in a highly dynamic fashion and argues that the ESs are not tethered within the nucleus.

If the ESs are not fixed to each other, statistically speaking, it would be unlikely that more than 2 ESs interact with each other at any one time. This is in agreement with the observation that, in cells with 3 ESs marked with drug resistance genes, only 2 of the 3 ESs are able to establish a rapid switching state (27). In addition, steric hindrance could prevent more than 1 invading ES from getting close enough to the ESB to sequester the components. The exact positioning of the ESs within the nucleus in relation to the ESB could therefore play a role in their relative frequency of activation, thereby determining the preferential hierarchy of ES activation observed earlier (51, 52).

Importantly, our ChIP analysis of epigenetic markers including histone H3, H1, base J, and TDP1 show that it is possible to have 2 ESs in the same cell that appear to have the epigenetic markers of fully active ESs, even though they are not both stably transcriptionally active. This is in agreement with results showing that, if ES transcription is inducibly silenced, the ES chromatin structure remains open for at least 24 h after induction of the transcription block (37). During this period, there is transient and reversible derepression of multiple silent ESs before commitment to 1 ES by 48 h. In our double-expresser trypanosomes, both unstably active ESs appear to have an epigenetic state that is equivalent to that observed on fully active ESs. The fact that trypanosomes released from drug-selection pressure invariably relapsed back to either BES1 or BES2 at approximately equivalent frequencies argues that establishing an active epigenetic state is an essential first step for ES activation. However, the limiting step in establishing monoallelic exclusion appears to be the ability of an ES with the epigenetic marks of an active state to associate with or nucleate an ESB, which can stably accommodate only a single ES.

Double-expresser trypanosomes are normally not observed in wild-type populations, and the frequency of generation of these *T. brucei* double-expresser strains was very low (<10^8^) (26). It is possible that these double-expresser strains have acquired genetic mutations that facilitate their ability to become double-expressers. We are currently investigating if double-expressers that have relapsed back to single expression are more able to establish the double-expresser phenotype compared with wild-type cells. If so, this would argue that 1 or more heritable changes have facilitated this disruption of monoallelic exclusion in our strains.

Nuclear architecture presumably plays a role in the monoallelic exclusion observed at *T. brucei* ESs as well as in the monoallelic exclusion operating at the *var* gene family of the malaria parasite *P*. *falciparum* (6). Here, only 1 of 60 *var* genes is activated in a mutually exclusive fashion, under the control of epigenetic factors including histone modifications (53, 54). Location of *var* genes at the nuclear periphery plays a key role in this control (55, 56). Both silent and active *var* loci are located within different zones of the nuclear periphery, whereby *var* activation requires escape from a perinuclear repressive center to an area of the nuclear periphery compatible with *var* gene activation (25). In contrast, in *T. brucei*, the ESB is not located preferentially at the nuclear periphery and does not obviously localize to one particular region of the nucleus. However, it still remains to be seen if the active ES preferentially interacts with other genomic regions within the nucleus.

Essential interchromosomal genomic interactions have been documented in the monoallelic exclusion operating at the >1,000 different mammalian olfactory receptor genes expressed within the sensory neurons of the nose (1). Here, large heterochromatic domains sequester the silent olfactory receptor gene clusters (57). However, within these inactive domains, interchromosomal hubs between different olfactory receptor enhancers form, which assemble over the 1 transcriptionally active olfactory receptor gene (58). These clustered enhancers contribute to the formation of olfactory receptor compartments, which then form a multichromosomal superenhancer within the nucleus (5).

No activating enhancer has yet been shown to play a role in ES activation. Similarly, inactive ESs have not been shown to obviously cluster within a single silencing compartment within the nucleus. DNA FISH experiments detecting a characteristic 50-bp simple sequence repeat extending for tens of kilobases upstream of all known ES promoters (59) have indicated that these are relatively dispersed within the nucleus (22, 60, 61). However, more recent genome-wide chromosome conformation capture (Hi-C) experiments have found that the *VSG*s at the silent ESs appear to interact with each other at a higher than average frequency, arguing that there is at least some clustering of these sequences within the nucleus (52). These interchromosomal interactions could play a role in control of ES transcription, as well as the DNA rearrangements at the ES telomeres involved in *VSG* switching. However, it is still not clear where the active ES is located within the context of these different chromosomal compartments.

In summary, our results provide insights into the monoallelic control of VSG expression in African trypanosomes. We show that, if we perturb monoallelic exclusion in double-expresser cells, we do not generate cells with additional ESBs. In contrast, we generate a rapidly switching state, whereby nascent transcripts from both ESs are only simultaneously observed within a 200-nm compartment containing the ESB. This argues that the ESB contains limiting factors for ES transcription and RNA processing, and the restriction of these components to a single ESB facilitates monoallelic exclusion. The 2 selected ESs appear to move in a highly dynamic fashion within the nucleus and are in close proximity to each other within the ESB only in the presence of simultaneous selection. These results highlight the importance of the ESB structure in restricting monoallelic expression in *T. brucei*.

## Methods

### Generation of *T. brucei* Strains.

Bloodstream-form (BF) *T*. *brucei* 427 was cultured in vitro according to ref. 62, and the cell lines generated were derived from the *T. brucei* “single marker” line (63). The *VSG221* ES-targeting construct (MK188) containing a puromycin/thymidine kinase (pur/TK) fusion gene and an eGFP gene was inserted into the active *VSG221* ES immediately upstream of the telomeric *VSG221* gene. The upstream target fragment of the MK188 construct contains 70-bp repeats from the *VSG221* ES, and the downstream fragment the *VSG221* cotransposed region, as described in ref. 30.

These cells were cultured on puromycin selection (0.2 µg mL^−1^) to maintain activation of the VSG221 ES, while thymidine kinase could be used as a negative selectable marker. After validation, the cell line was removed from puromycin selection for 48 h and subsequently diluted into media containing ganciclovir (4 µg mL^−1^) and plated in 96-well plates. Clonal ganciclovir-resistant cells were generated that had activated a different ES. The generated VSG switch variants were screened by immunofluorescence microscopy for loss of expression of GFP, and their protein lysates were analyzed by Western blotting to identify VSGV02 expressers. A VSGV02-expressing trypanosome clone was identified, which was subsequently transfected with the *VSGV02* ES-targeting MK234 construct. This resulted in the integration of the hygromycin resistance and mCherry genes immediately upstream of the telomeric *VSGV02* gene. The MK234 construct has an upstream target fragment containing the 70-bp repeat region from the *VSGV02* ES and a downstream target fragment with the *VSGV02* cotransposed region. All primers used for construct assembly are listed in *SI Appendix*, Table S1. PCR amplification of *VSGV02* ES-containing sequences was performed by using TAR clones, which were described previously (11, 64). This VSGV02-expressing cell line is referred to as *T. brucei* (V02+)PG-VHC. The VSG221 ES was subsequently selected for in these cell lines, resulting in its reactivation. This isogenic cell line is *T. brucei* (221+)PG-VHC.

### Generation and Analysis of Double-Expresser *T. brucei* Cell Lines.

The double-expresser *T. brucei* (DE) KW01 cell line was generated by selecting the VSG221-expressing (221+)PG-VHC line for resistance to both hygromycin (50 µg mL^−1^) and puromycin (0.8 µg mL^−1^). The DE BH03 cell line was generated by using the same approach, but using the VSGV02-expressing parental (V02+)PG-VHC line. Frequency of generation of DE lines was low (<10^−8^). DE cells lines were continuously maintained on double drug selection to maintain double expression. DE lines were regularly validated by using flow cytometry to monitor for simultaneous expression of the eGFP and mCherry genes. In addition, immunofluorescence microscopy was used to confirm expression of the appropriate VSG.

To simultaneously knock down eGFP and mCherry, a 405-bp fragment of each gene was amplified and these were fused together. Sense and antisense fragments of this fusion were then inserted into the pLew100-V5x:Pex11 stem loop construct (gift of Jay Bangs, University of Buffalo, Buffalo, NY) (65) by using Gibson assembly. This fluorescent protein (FP) RNAi construct was transfected into the *T. brucei* (221+)PG-VHC and DE KW01 cell lines, resulting in the (221+)PG-VHC FP-RNAi and DE KW01 FP-RNAi cell lines. Tetracycline induction of RNAi resulted in “defluorescing” the cells through simultaneous knockdown of both eGFP and mCherry.

To epitope-tag the N terminus of the RNA polymerase I (Pol I) RPA2 subunit (Tb927.11.630) with mNeonGreen, the coding sequence of mNeonGreen (mNG) was amplified from the pPOTv4-blast-mNG plasmid (66) and was used to replace eYFP in the pEnt5H-Y:NLS:RPA2 plasmid (both plasmids gift of the laboratory of Keith Gull, University of Oxford, Oxford, UK) (67) by using Gibson assembly. The hygromycin resistance gene was also replaced with a blasticidin resistance gene. The resulting pEnt5B-mNG::RPA2 plasmid was transfected into the (221+)PG FP-RNAi and DE KW01 FP-RNAi cell lines. For VEX1 tagging, cells were transfected with the pNATVEX1^x12myc^ construct (gift of David Horn, University of Dundee, Dundee, UK).

To detect nascent transcription using RNA-FISH, a 24× MS2 repeat sequence obtained from Addgene (plasmid no. 27120) (68) was inserted into the tubulin locus or BES2. To target the 24× MS2 sequence to the tubulin locus, the pTub-Hyg-117-VSG3UTR construct (69) was modified such that the Hyg-117 cassette was exchanged for the 24× MS2 sequence and the blasticidin resistance gene. This pTub-24×MS2-blast construct was transfected into the “single-marker” *T. brucei* cell line (63). For targeting MS2 repeats into BES2, the hygromycin and mCherry genes in the MK234 construct were exchanged for a blasticidin gene and a 24× MS2 repeat array. This 70-bp 24×MS2-V02CTR construct was transfected into (V02+)PG-VHC or DE KW01 FP-RNAi cells. Integration of the MS2 repeat array into BES2 resulted in the exchange of the hygromycin resistance and mCherry genes for the blasticidin resistance gene and the 24× MS2 repeat array. Activation of BES2 was then maintained by using selection on 20 µg^−1^ mL blasticidin. All primers used for construct assembly are listed in *SI Appendix*, Table S1.

Growth curves were performed, monitoring for cell proliferation, by using a Neubauer hemocytometer. Stability of the DE lines was monitored by removing the lines from double drug selection. Here, *T. brucei* cells were washed in prewarmed HMI-9 medium without drugs and resuspended in media that did or did not contain the relevant selection drugs. Cells were cultured, and flow cytometry analysis was performed as detailed later.

### Flow Cytometry and Protein Analysis.

*T. brucei* lines were fixed in 2% paraformaldehyde for 20 min in the dark at room temperature. Cells probed with anti-VSG antibodies were incubated in 0.5% BSA for 30 min and then incubated with anti-VSG221(-CRD) antibody (Jay Bangs, University of Buffalo, Buffalo, NY) or anti-VSGV02 antibody (Piet Borst, Netherlands Cancer Institute, Amsterdam, The Netherlands) for 1 h. Cells were washed with PBS solution before 1-h incubation with goat anti-rabbit IgG Alexa Fluor 647 secondary antibody. Cells were washed in PBS solution and analyzed by using an LSRFortessa analyzer (BD Bioscience) along with the appropriate compensation controls. Flow cytometry data were analyzed by using FlowJo V10 software (FlowJo). Compensation was applied by using the FlowJo software, and double-expresser cells were gated using the 98th percentile of the relevant controls.

For the Western blot analyses, protein was extracted from 10^7^ cells per cell line, and 5 × 10^5^ cell equivalents were loaded per lane. Gels were electrophoresed, blotted, and probed with anti-VSG221 antibody, anti-VSGV02 antibody, or anti-BiP (Jay Bangs, University of Buffalo, Buffalo, NY) by using standard protocols.

### Chromatin Immunoprecipitation.

Chromatin immunoprecipitation (ChIP) analysis of the distribution of histone H3, histone H1, and TDP1 was performed as previously described in ref. 70, with minor modifications. Cells were cross-linked with 1% formaldehyde for 1 h at room temperature. Cross-linking was quenched by the addition of glycine to a final concentration of 125 mM. Chromatin was sonicated (BioRuptor; Diagenode) to an average DNA fragment size of 200 bp, and the extract was clarified by centrifugation. Immunoprecipitation was performed for 18 h at 4 °C with the relevant antibody or no antibody as a negative control. Antibodies used were anti-histone H3 (Ab1791; Abcam), anti-histone H1 (described in ref. 30), and anti-TDP (described in ref. 36) coupled to Dynabeads Protein G magnetic beads (Novex; Life Technologies). ChIP experiments were performed in triplicate, and the resulting material was analyzed by qPCR using the 7500 Fast Real-Time PCR system (Life Technologies). qPCR primers were previously described in refs. 28, 69, and 70 or are listed in *SI Appendix*, Table S2. Quantitation of immunoprecipitated material was calculated as the percentage of input chromatin immunoprecipitated after subtraction of the value for the no-antibody control.

J-IP seq experiments were performed following a protocol modified from a previous work (35). Briefly, genomic DNA was sheared to ∼200 bp and immunoprecipitated by using an antibody raised against base J (34). Libraries were prepared from the J-IP and corresponding pre-IP samples sequenced (PE75) on an Illumina HiSeq 4000 system, and reads aligned against the *T. brucei* 427 reference genome (v36, with reconstructed BES1 and BES2 sequences) using Bowtie2. Statistical significance of the ChIP data were determined by using 2-way ANOVA with Tukey’s post hoc test and considered significant when *P* < 0.05 (*SI Appendix*, Table S5).

### Microscopy and Image Analysis.

For immunofluorescence microscopy, cells were washed in 1× PSG buffer before fixing in a final concentration of 2% paraformaldehyde (PFA) for 15 min. For VEX1 microscopy, VEX1::12myc-tagged cells were permeabilized with 0.1% Nonidet P-40 for 5 min, followed by 1 h blocking in 1% BSA. Slides were then incubated with an anti-myc antibody (4A6; EMD Millipore), followed by incubation with a goat anti-mouse secondary antibody. Fixed cells were mounted on slides by using Vectashield containing the DNA stain DAPI. Microscopy was performed by using a Zeiss Imager.M1 microscope equipped with a Zeiss AxioCam MRm camera and Axio Vision Rel 4.8 software. *Z*-stacks were taken in 0.2-µm increments. For VSG immunofluorescence microscopy, cells were fixed in 1% PFA for 10 min and blocked in 1% BSA for 1 h. Cells were incubated with a mouse anti-VSG221 antibody (gift of Nina Papavasiliou, Deutsches Krebsforschungszentrum, Heidelberg, Germany) and rabbit anti-VSGV02 antibody (gift of Piet Borst) for 1 h, followed by incubation with goat anti-mouse DyLight488 antibody and goat anti-rabbit DyLight594 antibody. Microscopy was performed by using a Zeiss AxioImager M2 microscope with an ORCA Flash 4 camera (Hamamatsu) and Zen software.

For superresolution structured illumination microscopy (SR-SIM), slides were sealed with Zeiss high-performance coverslips and imaged by using a Zeiss Elyra PS.1 with a sCMOS PCO Edge (SIM) camera using Zeiss Zen software. For image acquisition, *z*-stacks were taken in 0.1-µm intervals with 5 phases and 3 rotations using 0.28-µm grid spacing. Structured illumination reconstruction was performed by using Zen software, and SR-SIM data quality was assessed by using SIM check. Chromatic aberrations were corrected for by using 100-nm Tetraspeck beads (Thermo Fisher Scientific) with the channel alignment function in Zen software for each SR-SIM experiment. Channel alignment was determined in ImageJ for fluorescence microscopy. Quantitation of the distances between FISH signals was performed by taking a maximum-intensity projection of *z*-stacks and manually delineating a line profile between each focus of interest by using ImageJ. The fluorescence intensity output of each channel was then plotted, and the center of each focus of interest (as determined by the pixel with the greatest intensity) was used to determine the distance between them. Statistical analysis for microscopy data was performed by Student’s *t* test (paired, 2-tailed) unless stated otherwise. Data were considered significant when *P* < 0.05.

### FISH Experiments.

FISH analysis performed on cell lines containing the FP-RNAi construct was conducted at least 72 h after induction of RNAi by using 1 µg mL^−1^ tetracycline. For RNA FISH experiments, cells were fixed directly in medium for 15 min using by paraformaldehyde at a final concentration of 4%. Cells were washed in 1× PBS buffer and settled on microscopy slides (ThermoShandon) for 30 min. Cells were then permeabilized by immersing slides in 70% ethanol for 1 h in the presence of 25 U of RNase inhibitor (Roche). For combined RNA-FISH with mNG::RPA2 imaging, cells were instead permeabilized with 0.1% Nonidet P-40 for 5 min. Cells were then washed twice in 1× PBS solution and in RNA wash buffer (10% formamide, 2× SSC) for 5 min each. For detection of transcription from BES1, probe sets containing 40 Stellaris RNA-FISH probes of 20 nucleotides (nt; LGC Biosearch Technologies) were designed that were complementary to a *VSG* pseudogene (Tb427.BES40.21) present within BES1. These probe sets were conjugated to a C3-fluorescein dye or a Quasar-670 dye. To detect transcription from the MS2 repeats, 3 different Stellaris RNA-FISH probes (20 to 22 nt) were generated, which were complementary to the MS2 repeats, and were conjugated to Quasar-570 dye. All RNA-FISH probe sequences are listed in *SI Appendix*, Table S3.

Each probe was mixed to a final concentration of 125 nM in hybridization buffer (10% dextran sulfate, 10% formamide, 2× SSC). The probe solution was then preheated to 45 °C and added to samples, which were then sealed with Gene Frames (Thermo Fisher Scientific) and hybridized at 45 °C overnight in a hybridization oven. After hybridization, Gene Frames were removed, and samples were washed for 30 min at 45 °C with preheated RNA wash buffer. Slides were then washed for 5 min with 2× SSC and mounted with VectaShield containing DAPI (Vector Laboratories). All buffers were prepared using DEPC-treated water. Samples with combined RNA-FISH for VSGΨ and MS2 with mNG::RPA2 imaging were imaged by using a Hamamatsu ORCA-Flash4 with Zeiss Zen software. To measure half-lives of RNA-FISH fluorescent signals, before PFA fixation, cells were treated with 2.5 µg mL^−1^ sinefungin (Millipore) for 5 min followed by incubation with 10 µg mL^−1^ actinomycin D (Sigma) for 0.5, 1, 1.5, 2, 4, and 8 min. The percentage of cells with VSGΨ and MS2 signal was then quantitated and plotted by using the 1-phase exponential decay function in GraphPad.

For DNA FISH experiments, biotin- or digoxigenin-labeled DNA probes were generated by PCR using standard conditions with Taq polymerase, with the exception that a 1:2 ratio of biotin-16-dUTP (Sigma) or digoxigenin-11-dUTP (Sigma) and dTTP were used in the reaction. Fourteen to sixteen 139- to 282-bp fragments specific for the relevant single-copy fluorescent protein genes or drug resistance markers were amplified from plasmid DNA used to generate the DE cell lines. Fragments from BES1 or BES2 were amplified from TAR clones (11, 64). Primers used to generate the DNA-FISH probes are listed in *SI Appendix*, Table S4. All PCR fragments specific for BES1 were labeled with digoxigenin, whereas all fragments specific for BES2 were labeled with biotin. DNA probes were coprecipitated with salmon sperm DNA (Invitrogen) at 10 µg mL^−1^ and yeast tRNA (Sigma) at 10 µg mL^−1^. Probes were then resuspended to a concentration of 10 ng mL^−1^ in hybridization buffer (50% formamide, 10% dextran sulfate, 2× SSC).

Before hybridization, cells were prepared in the same manner as for RNA-FISH with the exception that permeabilization was performed with 0.1% Nonidet P-40 for 10 min followed by incubation with 50 µg mL^−1^ RNase (Sigma) for 1 h at 37 °C. After adding probe mix to slides, samples were sealed with Gene Frames and denatured on an inverted heat block at 85 °C for 5 min, followed by overnight incubation at 37 °C. After hybridization, slides were washed in DNA wash buffer (50% formamide, 2× SSC) for 30 min at 37 °C, followed by three 10-min washes in 1× SSC, 2× SSC, and 4× SSC at 50 °C. Samples were then incubated with an anti-digoxigenin antibody (Abcam 21H8) diluted 1:10,000 in 1% BSA (wt/vol in 1× PBS solution) for 1 h at 37 °C. After washing 3 times for 10 min in TBS with 0.01% Tween, slides were incubated for 45 min with a streptavidin-Alexa Fluor 488 conjugate (Thermo Fisher Scientific) and a goat anti-mouse Alexa Fluor secondary antibody (Thermo Fisher Scientific), both diluted to 1:500 in 1% BSA. Samples were washed in TBS with 0.01% Tween as before and mounted in Vectashield with DAPI.

## Supplementary Material

Supplementary File
