# Supplementary material for: Dynamic colocalization of 2 simultaneously active VSG expression sites within a single expression-site body in Trypanosoma brucei
Source: Proc Natl Acad Sci U S A. 2019 Jul 29;116(33):16561–70. doi: 10.1073/pnas.1905552116 (PMC6697882; doi:10.1073/pnas.1905552116)
Supplement: Supplementary File [file pnas.1905552116.sapp.pdf]

PNAS
www.pnas.org

Supplementary Information for

Dynamic co-localisation of two simultaneously active *VSG* expression sites within a single Expression Site Body in *Trypanosoma brucei*

James Budzak, Louise E. Kerry, Aris Aristodemou, Belinda S. Hall, Kathrin Witmer, Manish Kushwaha, Carys Davies, Megan L. Povelones, Jacqueline A. McDonald, Aakash Sur, Peter J. Myler and Gloria Rudenko

Gloria Rudenko
Email: gloria.rudenko@imperial.ac.uk

This PDF file includes:

Figs. S1 to S8
Tables S1 to S5

www.pnas.org/cgi/doi/10.1073/pnas.1905552116

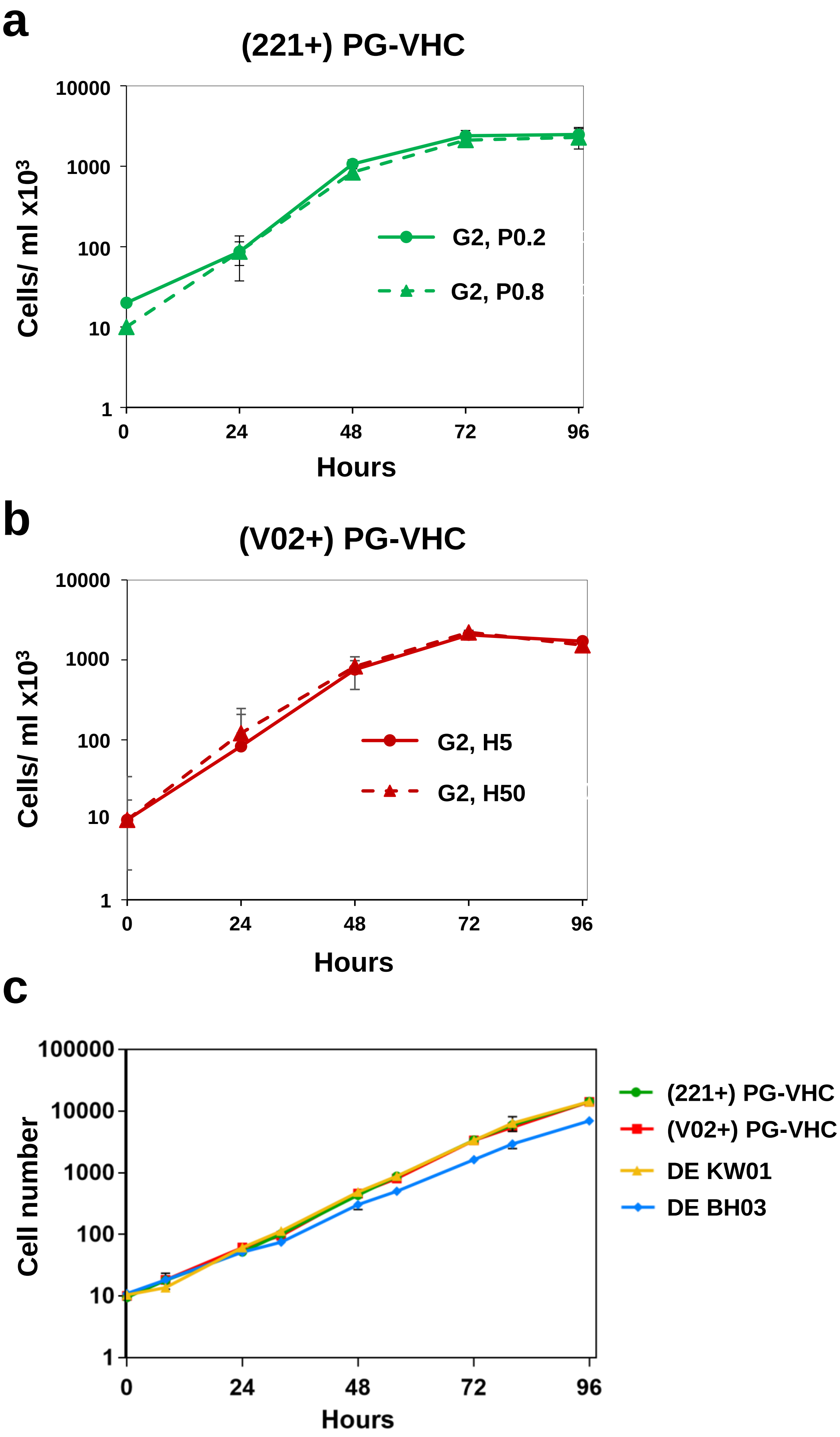

a
(221+) PG-VHC
Cells/ ml x10³
10000
1000
100
10
1
0
24
48
72
96
Hours
G2, P0.2
G2, P0.8
b
(V02+) PG-VHC
Cells/ ml x10³
10000
1000
100
10
1
0
24
48
72
96
Hours
G2, H5
G2, H50
c
Cell number
100000
10000
1000
100
10
1
0
24
48
72
96
Hours
(221+) PG-VHC
(V02+) PG-VHC
DE KW01
DE BH03

Sup Fig. S1

Sup. Fig. S1 Growth curves of parental single-expresser and double-expresser (DE) cell lines.

a. The (221+) PG-VHC cell line shows no significant growth defect when cultured in higher concentrations of puromycin (P) (0.8 µg ml^{-1} compared with 0.2 µg ml^{-1}). The concentration of G418 (G) remains constant at 2 µg ml^{-1}.

b. The (V02+) PG-VHC cell line shows no significant growth defect when cultured in higher concentrations of hygromycin (H) (50 µg ml^{-1} compared with 5 µg ml^{-1}). The concentration of G418 (G) remains constant at 2 µg ml^{-1}.

c. Growth curves of the DE KW01 and DE BH03 cell lines compared with the parental cell lines (221+) PG-VHC and (V02+) PG-VHC. Values shown are averages from 3 biological replicates. Error bars indicate ± SD.

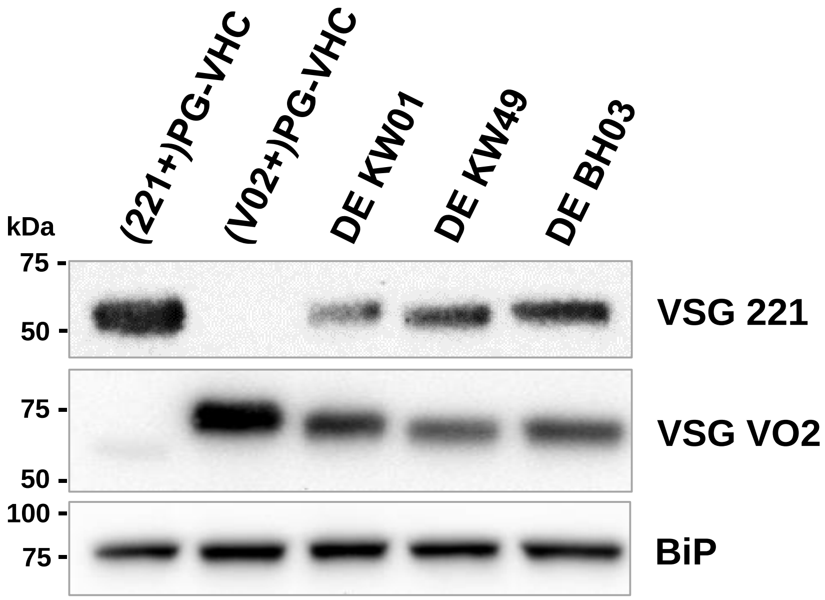

Sup. Fig. S2
The double-expresser DE KW01, DE KW49 and DE BH03 cell lines express approximately equivalent amounts of both VSG221 and VSGV02.

Western blot analysis of whole cell protein lysate extracted from the single-expresser cell lines (221+)PG-VHC and (V02+)PG-VHC and the double-expresser cell lines DE KW01, DE KW49 and DE BH03. Lysate from 5 x 10^5 cells was analysed in each lane, and the membrane was probed with antibodies against VSG221 and VSGV02. The endoplasmic reticulum protein BiP was used as a loading control. Size markers are indicated in kiloDaltons (kDa).

Sup Fig. S2

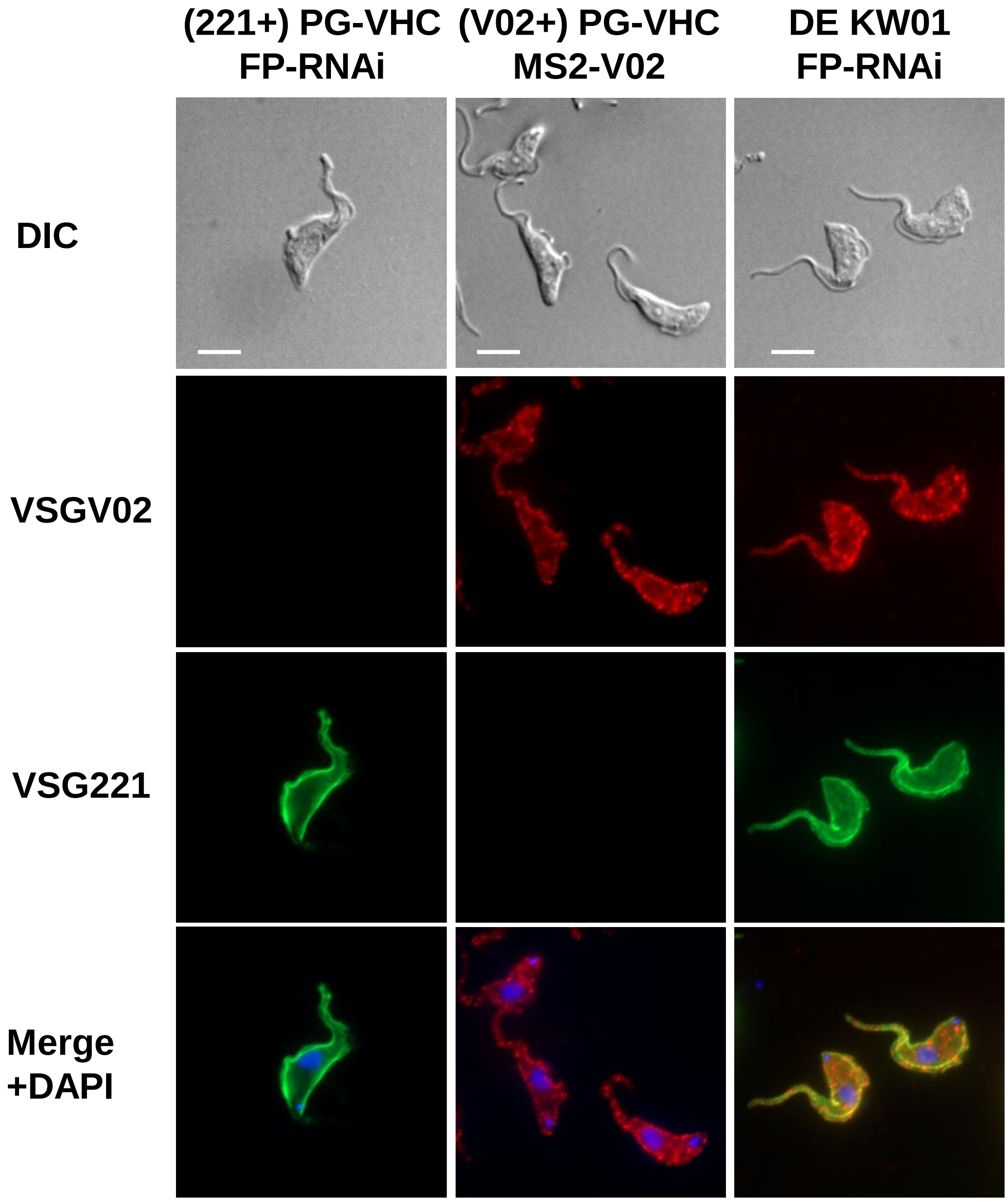

Sup. Fig. S3
Immunofluorescence microscopy analysis of VSG expression in single and double expresser cell lines.

Immunofluorescence microscopy analysis of single-expresser VSG221 expressing line (221+) PG-VHC FP RNAi and VSGV02 expressing line (V02+) PG-VHC MS-V02. The double-expresser cell line DE KW01 FP-RNAi expresses both VSG221 and VSGV02. Cells were incubated with anti-VSGV02 (red) and anti-VSG221 (green) antibodies. DNA was stained with DAPI (blue). FP-RNAi lines were treated with 1 µg ml^{-1} tetracycline 72 hours prior to microscopy. Scale bars represent 5 µm.

Sup Fig. S3

a

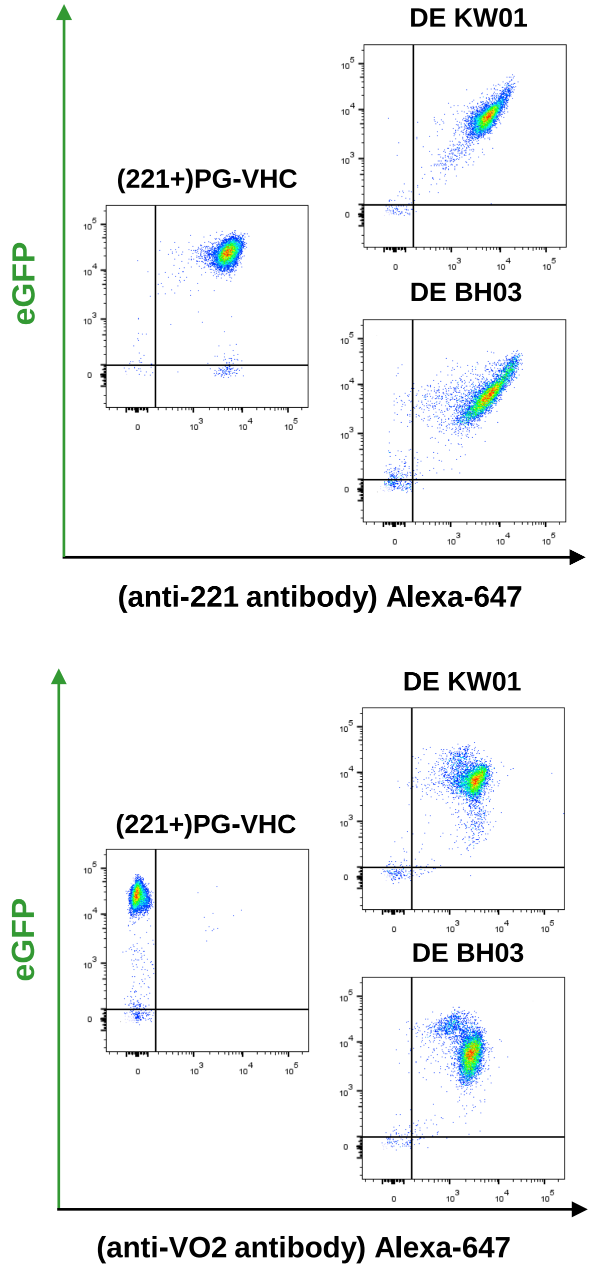

b

Endogenous fluorescence	VSG coat	Cell line			
		(221+)PG-VHC	(V02+)PG-VHC	KW01	BH03
GFP	221	98 %	-	99 %	95 %
	VO2	0 %	-	98 %	99 %
mCherry	221	-	3 %	99 %	99 %
	VO2	-	94 %	97 %	95 %

Sup Fig S4

Sup. Fig. S4

Expression of eGFP or mCherry located in either BES1 or BES2 respectively corresponds with expression of the corresponding BES VSG (VSG221 or VSGV02).

a. Flow cytometry traces of single-expresser (221+)PG-VHC or (V02+)PG-VHC lines compared with the double-expresser DE KW01 or BH03 lines. Fluorescence of either eGFP or mCherry is shown on the Y-axis. The cells were reacted with antibody against VSG221 or VSGV02 with signal indicated on the X-axis. Gates are set based on the 98th percentile of the relevant control.

b. The table shows the percentage (%) of cells within the eGFP or mCherry positive subpopulations which are also positive for staining with either anti-VSG221 or anti-VSGV02 antibodies.

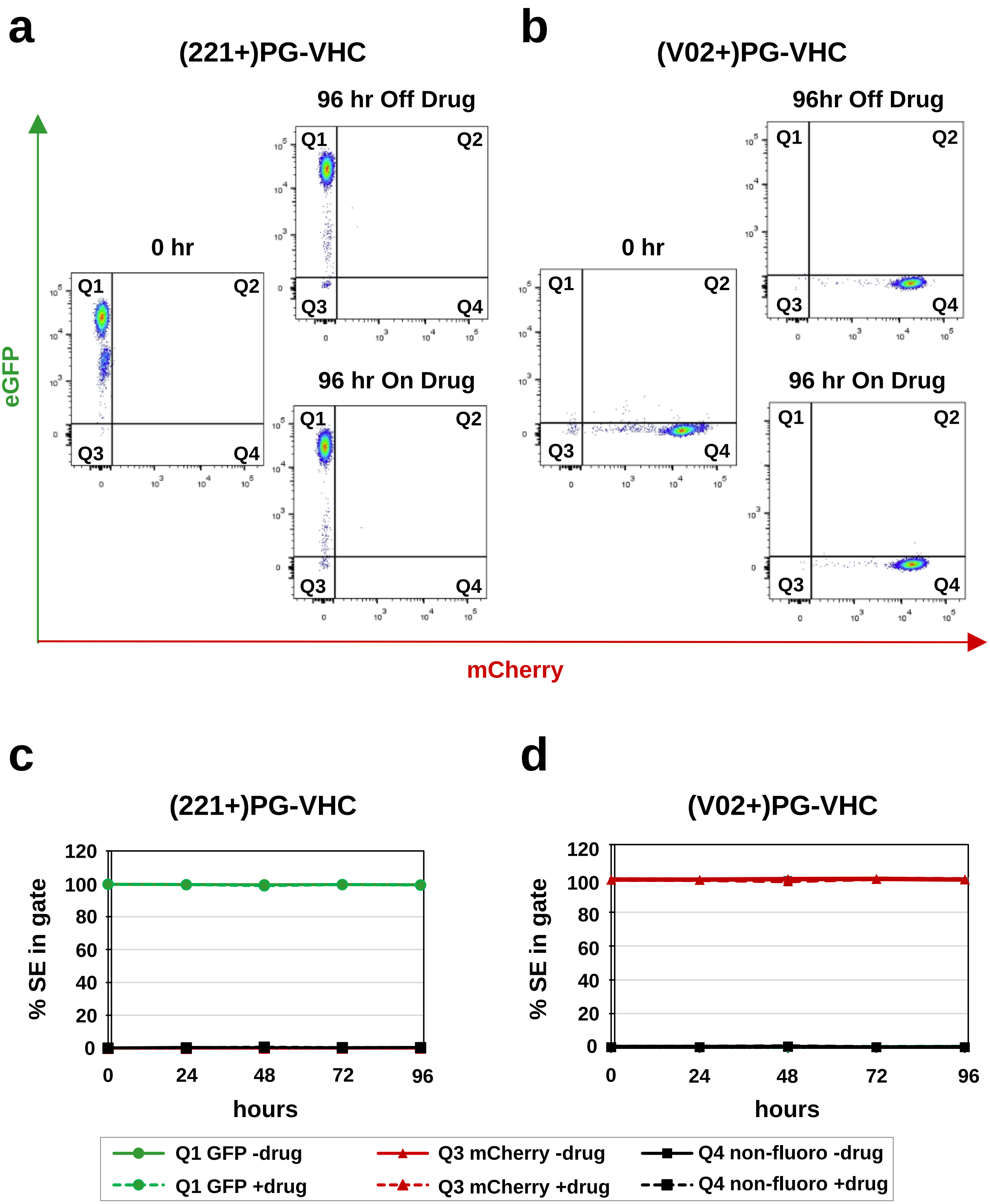
a
(221+)PG-VHC
0 hr
96 hr Off Drug
96 hr On Drug
b
(V02+)PG-VHC
0 hr
96hr Off Drug
96 hr On Drug
Q1
Q2
Q3
Q4
eGFP
mCherry
c
(221+)PG-VHC
d
(V02+)PG-VHC
% SE in gate
hours
Q1 GFP -drug
Q1 GFP +drug
Q3 mCherry -drug
Q3 mCherry +drug
Q4 non-fluoro -drug
Q4 non-fluoro +drug

Sup Fig. S5

Sup. Fig. S5

The single-expresser cell lines maintain stable expression of the relevant ES in the absence of drug selection for 96 hours (hr).

a. Representative flow cytometry traces of (221+)PG-VHC are shown either 0 or 96 hours in the presence (on drug) or absence (off drug) of drug selection. Quadrant gates (Q1-Q4) were determined using the 98th percentile.

b. As in a. but using the (V02+)PG-VHC single-expresser line.

c. The percentage (%) of single-expressers (SE) present in the single-expresser line (221+)PG-VHC after removal from drug selection for the time indicated are shown plotted. The percentage of cells in the Q1 (eGFP positive) vs nonfluorescent (Q4) quadrants are shown.

d. As in c., only the percentage of single-expressers in Q3 (mCherry positive) compared with those in Q4 are shown.

Histone H1

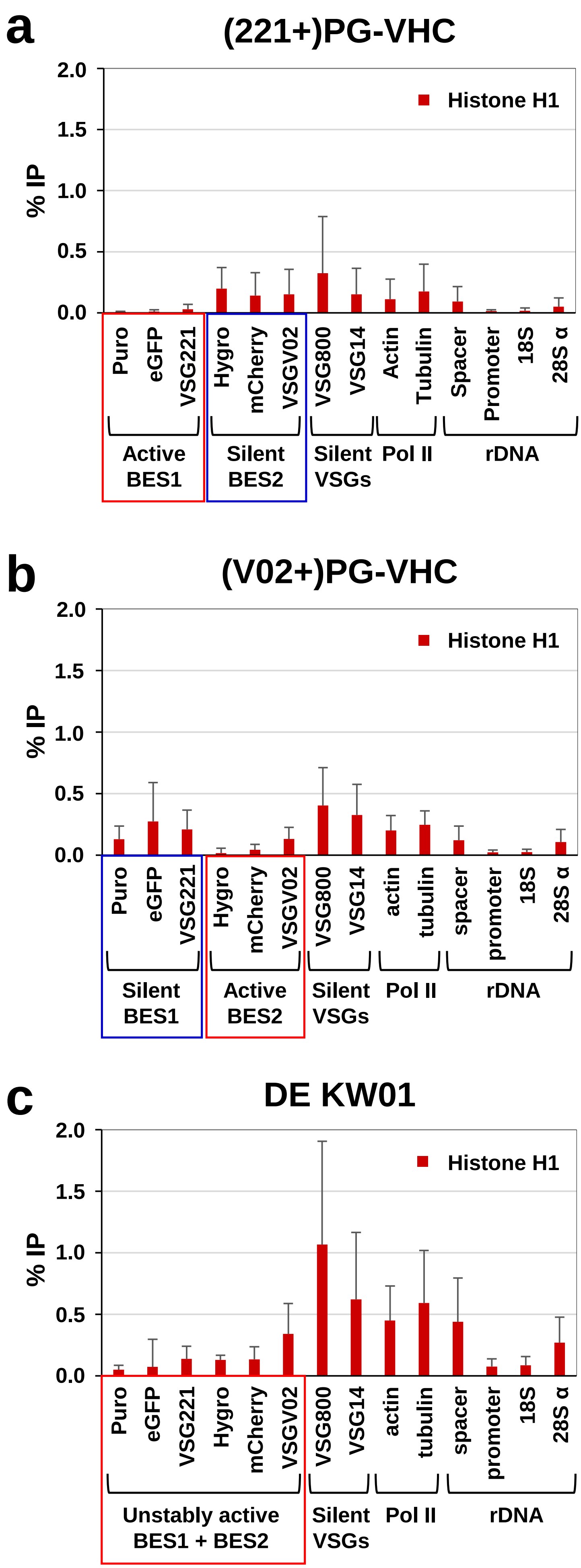

Sup Fig. S6

Sup. Fig. S6

Histone H1 is depleted from both stable and unstably active BESs.

a. The distribution of histone H1 was determined by ChIP using antibodies against histone H1 in the single-expresser (221+)PG-VHC cell line. qPCR analysis was performed detecting sequences present in either BES1 or BES2, as well as silent VSGs, genes present in Pol II transcription units as well as the rDNA. The mean percentage ± SD of input immunoprecipitated (% IP) after subtraction of the no antibody control is shown for three biological replicates. Sequences present in stable or unstably activated ESs are indicated with red boxes, and sequences from inactive ESs with blue boxes.

b. As in a. but for the single-expresser (V02+)PG-VHC line.

c. As in a. but for the double-expresser DE KW01 line.

TDP1

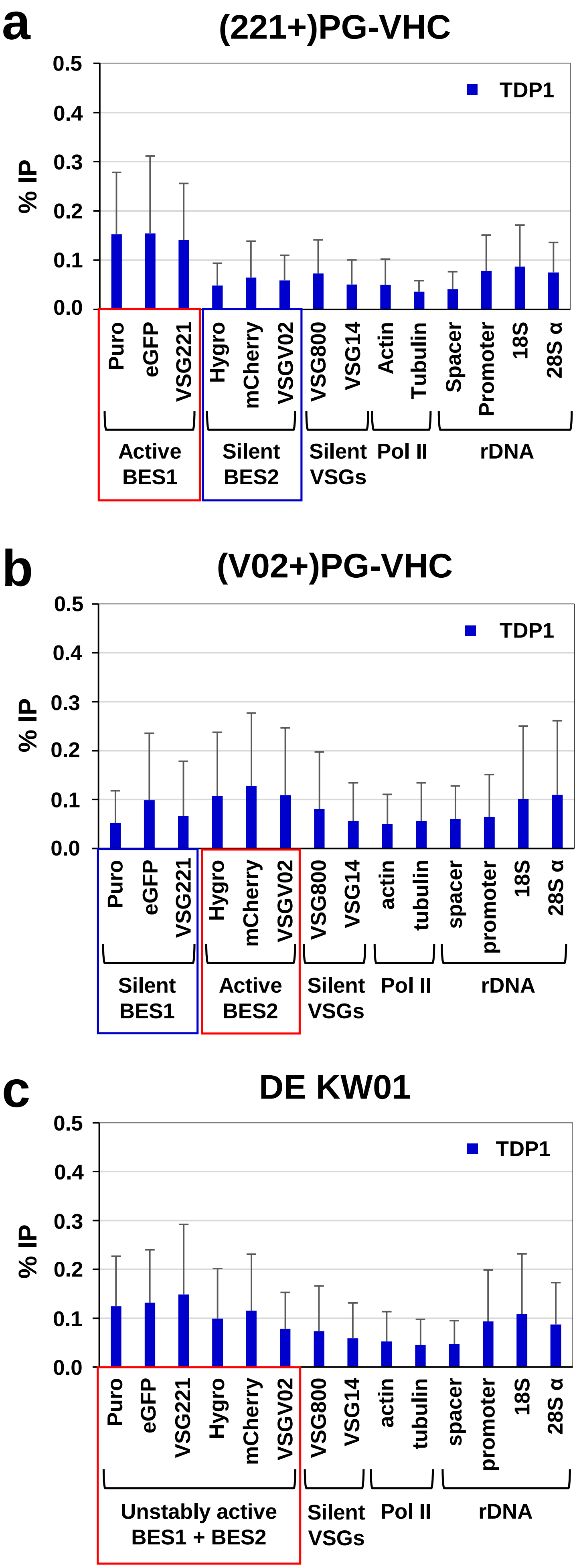

Sup Fig. S7

Sup. Fig. S7

The architectural chromatin protein TDP1 is enriched on stable and unstably active BESs.

a. The distribution of TDP1 was determined in the single-expresser (221+)PG-VHC cell line using chromatin immunoprecipitation (ChIP) with an antibody against TDP1. ChIP material was analysed by qPCR detecting sequences present in BES1 or BES2, as well as silent VSGs, genes present in Pol II transcription units as well as the rDNA. The mean percentage ± SD of input immunoprecipitated (%IP) after subtraction of the no antibody control is shown for three biological replicates. Sequences present in stable or unstably activated ESs are indicated with red boxes, and sequences from inactive ESs with blue boxes.

b. As in a. but for the single-expresser (V02+)PG-VHC line.

c. As in a. but for the double-expresser DE KW01 line.

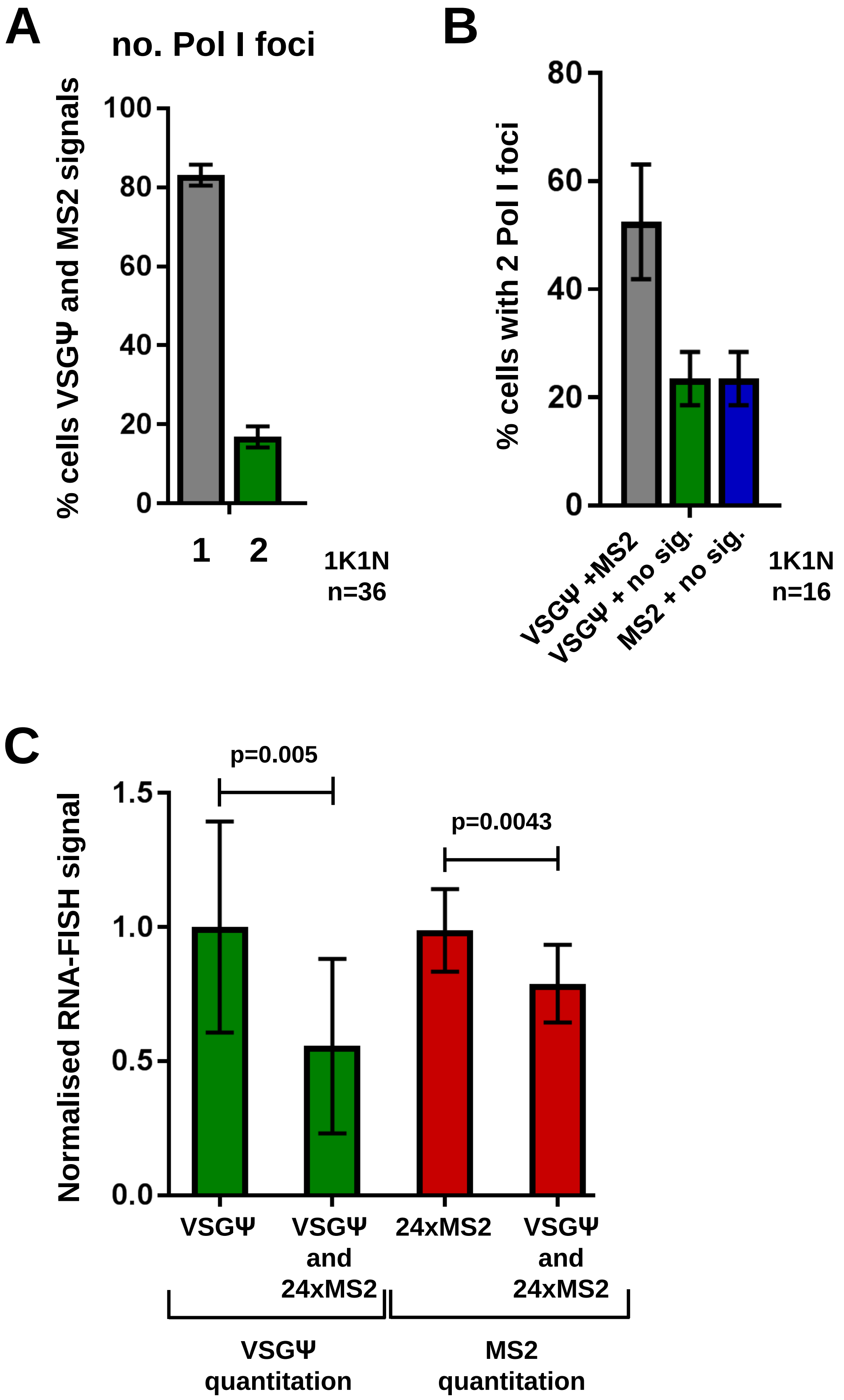
A
no. Pol I foci
% cells VSGΨ and MS2 signals
100
80
60
40
20
0
1
2
1K1N
n=36
B
% cells with 2 Pol I foci
80
60
40
20
0
VSGΨ +MS2
VSGΨ + no sig.
MS2 + no sig.
1K1N
n=16
C
Normalised RNA-FISH signal
p=0.005
p=0.0043
1.5
1.0
0.5
0.0
VSGΨ
VSGΨ and 24xMS2
24xMS2
VSGΨ and 24xMS2
VSGΨ quantitation
MS2 quantitation

Sup Fig. S8

Sup. Fig. S8

Simultaneous transcription of the unstably active ESs in double-expresser cells occurs predominantly in the same ESB. Data are shown for cells in G1.

a. Quantitation of the number of extra-nucleolar Pol I foci in DE KW01 cells which contain the RPA2 Pol I subunit epitope tagged with mNeon green and 24x MS2 repeats in BES2 (DE-KW01-MS2-V02 mNG::RPA2). The number of Pol I foci was determined in cells which contained signal for both VSGΨ (in BES1) and the 24x MS2 repeats (in BES2). Values shown are averages from two biological replicates, error bars indicated ± SD.

b. Cells with 2 Pol I foci in the DE KW01-MS2-V02 mNG::RPA2 strain were analysed, and it was determined if signal for BES1 (VSGΨ) or BES2 (MS2) was present in either or both foci. Values shown are averages from two biological replicates, error bars indicate ± SD.

c. The DE KW01 MS2-V02 cell line was analysed, and the amount of signal for either VSGΨ or MS2 nascent transcript was quantitated using RNA-FISH. VSGΨ or MS2 repeat signal intensities were normalised against the average value of either VSGΨ or MS2 repeat signal respectively. Values shown are the average of three biological replicates ± SD. At least 29 cells were counted for each measurement.

Primer name	Primer sequence (5'-3')	Template	Amplified product
plew-mCherry_s	accaaaaagtaaaattcacaCGAGGGCGAGGGCGAGGG	DE KW01 gDNA	mCherry RNAi fragment
eGFP-mCherry_as	gggcagcttgCTCTGCTTGATCTCGCCCTTCAGGG	DE KW01 gDNA	
mCherry_eGFP_s	tcaagcagagCAAGCTGCCCGTGCCCTG	DE KW01 gDNA	eGFP RNAi fragment
pLew-eGFP_as	cggggagggcgcgccttttcGTGTTCTGCTGGTAGTGGTCGG	DE KW01 gDNA	
pLew_mCh_eGFP_s	accaaaaagtaaaattcacaCGAGGGCGAGGGCGAGGG	DE KW01 gDNA	mCherry-eGFP chimera sense RNAi fragment
mCh-eGFP_as-HP	agcagaacacGGGCTTGAGTCGTAGCCAG	DE KW01 gDNA	
HP-mCherry-eGFP_s	actcggggagGTGTTCTGCTGGTAGTGGTCGGC	DE KW01 gDNA	mCherry-eGFP chimera antisense RNAi fragment
pLew-mCh-eGFP_as	cggggagggcgcgccttttcCGAGGGCGAGGGCGAGGG	DE KW01 gDNA	
HP-mCh-eGFP_as	actcaagcccGTGTTCTGCTGGTAGTGGTCGGC	pLEW100v5x:Pex11	Pex 11 hairpin
mCherry-eGFP-HP_as	agcagaacacCTCCCCGAGTGGACCAGG	pLEW100v5x:Pex11	
mNG_F_HindIII	ACTCAACTGCAACGAAGCTTatgggttctggtagtggttc	pPOT-v4-blast-mNG	mNG-CDS
mNG_R_HindIII	ACAACGGAATTCTTTGTTGAActacccgatcctgatccag	pPOT-v4-blast-mNG	
RPA2_CDS_F_XhoI	tcaacaaagaattccgttgt	S16 gDNA	RPA2 CDS
RPA2_CDS_R_Xho1	ACCTCACAACTGGGCTCGAGaataatggtcgatgcctctc	S16 gDNA	
infVO2ctr48323s	TTTTGAATCTTAATTTTTCGGGATCCGGAGGAGAAGGCCAGAAAAC	TAR129	V02 CTR target fragment
infVO2ctr48727as	GCTGGAGCTCCACCGCGGTGGCGGCCGCGAGCGCACGCTAGTTAGGTT	TAR129	

Supplementary Table 1. Primers used for cloning

Primer name	Primer sequence (5'-3')	Amplified product
GammaTub_491s	AATCACCGTATTTGGACACAAG	Y-Tubulin
GammaTub_583as	GTTTGAGTGTTCGCTGAAAGAG	
polI-349s	ATTCACCGTATTTGGACACAAG	Pol I
polI-486as	GTTTGAGTGTTCGCTGAAAGAG	
Actin_1031s	GTTCCATCCTCTCATCACTA	Actin
Actin_486as	TCGTATTCACTCTTCGTTATC	
VSG14_959s	CACTAGACTCAAATCACAAATC	VSG14
VSG14_1090as	GGTTTATCACTAGGTTGTCTT	
mCherry_528s	CACTACGACGCTGAGGTCAA	mCherry
mCherry_627s	GTGGGAGGTGATGTCCAACT	
eGFP_184s	GTGACCACCCTGACCTAC	eGFP
eGFP_324as	CTTGTAGTTGCCGTCGTC	
Hygro_419s	GAATCGGTCAATACACTACAT	Hygro
Hygro_492as	AGTTTGCCAGTGATACACAT	
VSG221_s4	GCGACAACCAGCCAACCAAG	VSG221
VSG221_as4	TCAGCGGGCTTGTGCTTCTG	
Puromycin_288s	CGAGTTGAGCGGTTCC	Puro
Puromycin_333as	GCCTTCCATCTGTTGCT	
VSGVO2_94s	CTAGTAGCCTCACAACGATG	VSGVO2
VSGVO2_199as	ACAGCCGCTGTATCTCTTGC	
rDNAspacer_178s	ATTTTCTCTACCCCTCTCTT	rDNA spacer
rDNAspacer_323as	ATCATCGTATCATTTTCATC	
rDNAprom_s	GTACGGAGCAGGAGAGCAAC	rDNA promoter
rDNAprom_as	GCATTGCGCAAAGTTTACAG	
18s_177s	GCATTACTGGATAACTTGG	18S rRNA
18S_250as	GTTCTAATTTCATTCATTCG	
28Salpha_226s	ACACATTTACAACCCTTCAT	28S alpha rRNA
28Salpha_410as	CTATCGGTCTTCCTACTCTAT	

Supplementary Table 2. Primers used for ChIP-qPCR

RNA-FISH probe name	Sequence (5'-3')
MS2 probe 1	tacggtacttattgccaagaaa
MS2 probe 2	tccagtattccagggttcatca
MS2 probe 3	ctccaggtcgaatcttcaaa
VSG_pseudogene_1	gcattaaattcggcgctgtt
VSG_pseudogene_2	cagcgctagtacgttgcata
VSG_pseudogene_3	cgcaactgctagaggcaaag
VSG_pseudogene_4	aattactgcattgcctttgc
VSG_pseudogene_5	tttctgtgcttgttgatata
VSG_pseudogene_6	ccgtcttttgagttgtagta
VSG_pseudogene_7	tttgtttctgttgtgtgtcg
VSG_pseudogene_8	tgtaatttggtagcccaatc
VSG_pseudogene_9	tgcgtcggctttatgaattt
VSG_pseudogene_10	tgaatttaatgatgccggtt
VSG_pseudogene_11	tgtttgctgcggtctagacg
VSG_pseudogene_12	ctgttgattagcctgatcac
VSG_pseudogene_13	ccttgcgttggatagttatc
VSG_pseudogene_14	ccttcaacttctctggtaat
VSG_pseudogene_15	aatagcgcgtcattcagttt
VSG_pseudogene_16	gaattcgttctttcctgttc
VSG_pseudogene_17	ccgcatatttggttttctac
VSG_pseudogene_18	gttcaataatggcccgtctg
VSG_pseudogene_19	cgacgcgaagacagtacaga
VSG_pseudogene_20	caaaggttggttggtgtgtc

VSG_pseudogene_21	ataattttgttgagctccgc
VSG_pseudogene_22	tttgtcgacgttgcattctg
VSG_pseudogene_23	gtaggccgttaatttcttga
VSG_pseudogene_24	atctgctgagcaaacctttt
VSG_pseudogene_25	ctggtagttgacgcacatgc
VSG_pseudogene_26	taccgcttttgcttagttga
VSG_pseudogene_27	tctgctagtcatttgattcc
VSG_pseudogene_28	ttcaaggctcgtctcaagaa
VSG_pseudogene_29	cccaggctgttgtaagtaaa
VSG_pseudogene_30	tttatctttggctgccgttg
VSG_pseudogene_31	tcttttggtctgtgttgtca
VSG_pseudogene_32	cttttccgttgttcgcattc
VSG_pseudogene_33	attttcgcatgtagttatgt
VSG_pseudogene_34	cattccatttgcatttacct
VSG_pseudogene_35	ttcatctttgggtttgcatt
VSG_pseudogene_36	tgttgctgtgtttggctgtt
VSG_pseudogene_37	ctttttgccttctgcatttg
VSG_pseudogene_38	cctcttctttttacttatcg
VSG_pseudogene_39	catttgcatccatctttgta
VSG_pseudogene_40	gaatctttgcaagtttcgcc

Supplementary Table 3. Sequences of RNA-FISH probes used in this study

Primer name	Primer sequence (5'-3')	Template	Amplified product
Hygro_1_s	AAAAAGCCTGAACTCACCGC	DE KW01 gDNA	Hygromycin probe 1
Hygro_1_as	CCCAATGTCAAGCACTTCCG	DE KW01 gDNA	
Hygro_2_s	TGACCTATTGCATCTCCCGC	DE KW01 gDNA	Hygromycin probe 2
Hygro_2_as	GCTCATCGAGAGCCTGCG	DE KW01 gDNA	
Hygro_3_s	GAAGTCCGGCACCTCGTG	DE KW01 gDNA	Hygromycin probe 3
Hygro_3_as	CGCGTCTGCTGCTCCATA	DE KW01 gDNA	
Hygro_4_s	CATCCGGAGCTTGCAGGA	DE KW01 gDNA	Hygromycin probe 4
Hygro_4_as	TTCCTTTGCCCTCGGACG	DE KW01 gDNA	
mCherry_1_s	AGCAAGGGCGAGGAGGATA	DE KW01 gDNA	mCherry probe 1
mCherry_1_as	GGTGCTTCACGTAGGCCTTG	DE KW01 gDNA	
mCherry_2_s	ACTTGAAGCTGTCCTTCCCC	DE KW01 gDNA	mCherry probe 2
mCherry_2_as	TGCTTGATCTCGCCCTTCAG	DE KW01 gDNA	
mCherry_3_s	GCCACTACGACGCTGAGG	DE KW01 gDNA	mCherry probe 3
mCherry_3_as	TTGTACAGCTCGTCCATGCC	DE KW01 gDNA	

V02_1_s	TGCCTCACACCAACGTAGAA	TAR129	V02 probe 1
V02_1_as	CTGTTGCTGCGTCAGGCG	TAR129	
V02_2_s	CAGCGGCTGTACATACTAACA	TAR129	V02 probe 2
V02_2_as	TTCAGTTGCTGTGCTTTCGT	TAR129	
V02_3_s	AAACTGATACGCCAAGCAACG	TAR129	V02 probe 3
V02_3_as	GGTTCCGGCGCTGCAAAG	TAR129	
V02_4_s	CAGACAAGCGCGAAAATGTG	TAR129	V02 probe 4
V02_4_as	GCGAGAATTCCCCTTTGTCAC	TAR129	
V02_5_s	GAGGCTGCGACGGAACAG	TAR129	V02 probe 5
V02_5_as	GGGTGTAGCGTTGCTGTGTA	TAR129	
V02_6_s	GACAGCGGGCGCAAATAC	TAR129	V02 probe 6
V02_6_as	CCATCTTTACCCTTTCTCCATGC	TAR129	
V02_7_s	AGGAAAAGTGCCGAAACGGT	TAR129	V02 probe 7
V02_7_as	AGTAGCAAGAATTTTTAGCAAGGAA	TAR129	
221_1_s	CCTTCCAATCAGGAGGCCC	TAR40	221 probe 1
221_1_as	ATACTTGCTCGCAGTGCCG	TAR40	
221_2_s	GCACCAACAGGGCCAAGG	TAR40	221 probe 2
221_2_as	GCCGGCCTTCGTTACCGT	TAR40	
221_3_s	GAGGCGTTCCTTGCAAGCTG	TAR40	221 probe 3
221_3_as	GCTGTTTGGCTGTTCGCTAC	TAR40	
221_4_s	GCGCTCGATGCATTACAGGA	TAR40	221 probe 4
221_4_as	GTTCATTTTCGTGGCCGCT	TAR40	
221_5_s	TCATATCCGAGCAGCCAGTG	TAR40	221 probe 5
221_5_as	TGCAGCTGCCTTCTGTTTCT	TAR40	
221_6_s	GAGTGCAAATCCCCATGCAA	TAR40	221 probe 6
221_6_as	AGCAAAACTGCAAGCCAAAGA	TAR40	
PurTK_1_s	ATGACCGAGTACAAGCCCAC	DE KW01 gDNA	PURTK probe 1

PurTK_1_as	CTCTCCGGCGTGGTCCAG	DE KW01 gDNA	PURTK probe 1
PurTK_2_s	GGCGGTGTTCGCCGAGAT	DE KW01 gDNA	PURTK probe 2
PurTK_2_as	AGGTCTCCAGGAAGGCGG	DE KW01 gDNA	
PurTK_3_s	ACCTCCCCTTCTACGAGCG	DE KW01 gDNA	PURTK probe 3
PurTK_3_as	CCGTACGTCGGTTGCTATGG	DE KW01 gDNA	
PurTK_4_s	AGCAAGAAGCCACGGAAGTC	DE KW01 gDNA	PURTK probe 4
PurTK_4_as	CGGCCGATATCTCACCCTG	DE KW01 gDNA	
PurTK_5_s	TGACAAGCGCCCAGATAACA	DE KW01 gDNA	PURTK probe 5
PurTK_5_as	ATGTTGGTGCCGGGCAAG	DE KW01 gDNA	
PurTK_6_s	GGAGGACAGACACATCGACC	DE KW01 gDNA	PURTK probe 6
PurTK_6_as	GGGCCCGAAACAGGGTAAAT	DE KW01 gDNA	
PurTK_7_s	ACGGCGACCTGTATAACGTG	DE KW01 gDNA	PURTK probe 7
PurTK_7_as	TTAGCCTCCCCCATCTCCC	DE KW01 gDNA	
GFP_1_s	GTGAGCAAGGGCGAGGAG	DE KW01 gDNA	GFP probe 1
GFP_1_as	GCTTCATGTGGTCGGGGTAG	DE KW01 gDNA	

GFP_2_s	TTCAAGTCCGCCATGCCC	DE KW01 gDNA	GFP probe 2
GFP_2_as	AGTTCACCTTGATGCCGTTCT	DE KW01 gDNA	
GFP_3_s	AACATCGAGGACGGCAGC	DE KW01 gDNA	GFP probe 3
GFP_3_as	TTGTACAGCTCGTCCATGCC	DE KW01 gDNA	

Supplementary Table 4. Primers used for generation of DNA-FISH probes

Base J ChIP-seq statistics

Comparison	Significance	Adjusted P Value
**VSGΨ**		
221+ vs. V02+	No	0.8549
221+ vs. KW01	No	0.9661
V02+ vs. KW01	No	0.9563
**purTK**		
221+ vs. V02+	No	0.3429
221+ vs. KW01	No	0.9891
V02+ vs. KW01	No	0.417
**eGFP**		
221+ vs. V02+	Yes **	0.0048
221+ vs. KW01	No	0.9748
V02+ vs. KW01	Yes **	0.0027
**VSG221**		
221+ vs. V02+	Yes ****	<0.0001
221+ vs. KW01	No	0.9959
V02+ vs. KW01	Yes ****	<0.0001
**hygro**		
221+ vs. V02+	No	0.861
221+ vs. KW01	No	0.9241
V02+ vs. KW01	No	0.9887
**mCherry**		
221+ vs. V02+	No	0.7649
221+ vs. KW01	No	0.7986
V02+ vs. KW01	No	0.9981
**VSGV02**		
221+ vs. V02+	No	0.7805
221+ vs. KW01	No	0.8109
V02+ vs. KW01	No	0.9984
**VSG800**		
221+ vs. V02+	No	0.094
221+ vs. KW01	No	0.1375
V02+ vs. KW01	No	0.9791
**VSG-14**		

221+ vs. V02+	Yes	****	<0.0001
221+ vs. KW01	No		0.1018
V02+ vs. KW01	Yes	**	0.0096
**γ-tubulin**			
221+ vs. V02+	No		0.9926
221+ vs. KW01	No		0.9995
V02+ vs. KW01	No		0.9959
**actin**			
221+ vs. V02+	No		>0.9999
221+ vs. KW01	No		>0.9999
V02+ vs. KW01	No		0.9998
**polI**			
221+ vs. V02+	No		0.9949
221+ vs. KW01	No		0.9963
V02+ vs. KW01	No		0.9999
**rDNA spacer**			
221+ vs. V02+	No		0.1401
221+ vs. KW01	No		0.8
V02+ vs. KW01	No		0.3954
**rDNA promoter**			
221+ vs. V02+	Yes	****	<0.0001
221+ vs. KW01	Yes	*	0.0162
V02+ vs. KW01	Yes	**	0.0088
**18S**			
221+ vs. V02+	No		0.9972
221+ vs. KW01	No		>0.9999
V02+ vs. KW01	No		0.9965
**28Sa**			
221+ vs. V02+	No		0.97
221+ vs. KW01	No		0.9912
V02+ vs. KW01	No		0.9935

Histone H3 ChIP statistics

**Comparison**	**Significance**	**Adjusted P Value**
**eGFP**		
221+ vs. V02+	Yes **	0.0011
221+ vs. KW01	No	0.9343
V02+ vs. KW01	Yes **	0.0036
**PurTK**		
221+ vs. V02+	Yes *	0.0291
221+ vs. KW01	No	0.9692
V02+ vs. KW01	No	0.0527
**VSG 221**		
221+ vs. V02+	No	0.1412
221+ vs. KW01	No	0.9677
V02+ vs. KW01	No	0.2231
**Hygro**		
221+ vs. V02+	No	0.0824
221+ vs. KW01	No	0.1659
V02+ vs. KW01	No	0.9391
**mCherry**		
221+ vs. V02+	Yes *	0.0175
221+ vs. KW01	Yes *	0.0469
V02+ vs. KW01	No	0.9228
**VSG V02**		
221+ vs. V02+	No	0.4336
221+ vs. KW01	No	0.8914
V02+ vs. KW01	No	0.7152
**VSG 800**		
221+ vs. V02+	No	0.9999
221+ vs. KW01	No	0.3095
V02+ vs. KW01	No	0.3168
**VSG 14**		
221+ vs. V02+	No	>0.9999
221+ vs. KW01	No	0.9414
V02+ vs. KW01	No	0.944
**Actin**		

221+ vs. V02+	No	>0.9999
221+ vs. KW01	No	0.4599
V02+ vs. KW01	No	0.4577
**PolI**		
221+ vs. V02+	No	0.7235
221+ vs. KW01	No	0.1916
V02+ vs. KW01	No	0.5884
**Y-Tubulin**		
221+ vs. V02+	No	0.9364
221+ vs. KW01	No	0.4867
V02+ vs. KW01	No	0.7017
**rDNA spacer**		
221+ vs. V02+	No	0.997
221+ vs. KW01	No	>0.9999
V02+ vs. KW01	No	0.9965
**rDNA promoter**		
221+ vs. V02+	No	>0.9999
221+ vs. KW01	No	0.9878
V02+ vs. KW01	No	0.9862
**18S**		
221+ vs. V02+	No	0.9766
221+ vs. KW01	No	0.9752
V02+ vs. KW01	No	0.9072
**28S alpha**		
221+ vs. V02+	No	0.989
221+ vs. KW01	No	0.8718
V02+ vs. KW01	No	0.9319

Supplementary Table 5. Statistical analysis of ChIP-seq for Base J and ChIP for Histone H3 in (221+) PG-VHC, (V02+) PG-VHC and DE KW01 cell lines.

Statistical significance of ChIP data was determined using two-way ANOVA and Tukey's post-hoc test and considered significant when $P<0.05$. *$P<0.05$, **$P<0.01$, ***$P<0.001$, ****$P<0.0001$.
